# Measurement of volatile organic compounds using tethered balloons in a polluted industrial site in Catalonia (Spain)

**DOI:** 10.1007/s11356-024-34020-3

**Published:** 2024-06-26

**Authors:** Isabel Díez-Palet, Clara Jaén, Esther Marco, Barend L. Van Drooge, Pilar Fernández, Joan O. Grimalt

**Affiliations:** 1 Institute of Environmental Assessment and Water Research (IDAEA-CSIC), Jordi Girona, 18-26, 08034 Barcelona, Catalonia Spain; 2 Faculty of Chemistry, University of Barcelona, Martí i Franquès 1-11, 08028 Barcelona, Catalonia Spain

**Keywords:** VOCs, Air pollution, Emission sources, Mixing layer, Tethered balloon sampling, GC-MS analysis

## Abstract

Understanding the chemical composition of volatile organic compounds (VOCs) near emission sources and in the background atmosphere above the mixing layer height (MLH) provides insight into the fate of VOCs and is essential for developing effective air pollution control strategies. Unfortunately, knowledge of the qualitative and quantitative changes of VOCs and their vertical transport in the atmosphere is limited due to challenging experimental setups. In this study, an innovative method using tethered balloons was tested and implemented to sample 40 VOCs and O_3_ below and above the MLH at an industrial site in Spain. VOC and O_3_ samples were collected with different types of sorbent cartridges and analyzed using chromatographic techniques. Overall, a decrease in VOC concentration with altitude was observed along with a homogeneous chemical composition up to 300 m AGL. This decrease with altitude denoted the primary origin of these VOCs, which were strongly influenced by industrial processes and the traffic emissions in the area. Conversely, O_3_ concentrations were notably higher at balloon level and increased during nighttime temperature inversion episodes in those samples collected above the mixing layer. Ground samples contained freshly emitted pollutants of industrial origin, while balloon samples consisted of aged pollutants from traffic, other combustion sources, or from a secondary origin. This study is the first to assess the vertical composition of VOCs at a site of these characteristics and demonstrates that tethered balloons are a cost-effective method for studying air pollution dynamics from the ground to higher altitudes in the low troposphere.

**Supplementary Information:**

The online version contains supplementary material available at 10.1007/s11356-024-34020-3.

## Introduction

 Air pollution is a topic of major concern since is one of the most pressing environmental issues for human health. According to the World Health Organization (WHO) in 2022, outdoor air pollution caused an estimated 4.2 million premature deaths worldwide in 2019. The main factor influencing human exposure to air pollutants is proximity to the emission sources. Consequently, densely populated urban and industrial areas are the environments with the highest population exposure. Besides direct emissions, stagnant atmospheric conditions, such as atmospheric temperature inversions, foster the accumulation of pollutants near the surface and exacerbate this exposition (Trinh et al. 2019).

Volatile organic compounds (VOCs) are an important class of air pollutants which are released from biogenic and anthropogenic sources. Despite biogenic VOCs represent the major fraction emitted worldwide (Guenther et al. 1995), anthropogenic compounds can dominate in urban and industrial areas (Atkinson and Arey 2003; Borbon et al. 2013; Gao et al. 2023). Furthermore, as industrial infrastructures and emission control policies evolve, solvent use and industrial processes are gradually replacing transport as the main VOC emission source (Wei et al. 2014; Zheng et al. 2017). The reactions of VOCs with atmospheric oxidants such as nitrogen oxides, hydroxyl radicals, and ozone (Atkinson and Arey 2003) contribute to the formation of secondary pollutants in the troposphere such as ozone or secondary organic aerosols and can be responsible for the formation of the photochemical smog layer over cities (Rani et al. 2011). Furthermore, some of these compounds are known to be carcinogenic to humans and animals (Kamal et al. 2016). In particular, benzene and formaldehyde are considered some of the most toxic VOCs. The former is a primarily emitted compound originated mostly from road traffic and is regulated in the EU air quality directive (2008/EC/50) with an annual limit concentration of 5 μg/m^3^. The latter may have a primary industrial origin as well as secondary formation through photochemical reactions involving high reactive VOCs (Parrish et al. 2012). In addition to health impact near to their primary sources, certain compounds with low photochemical reactivity can be subject to long-range atmospheric transport and act as contaminants in background areas (Simpson et al. 2020). Due to this complexity, it is essential to understand the chemical composition of these compounds not only near the sources at ground level, but also in background atmospheres, such as the free troposphere, above the mixing layer height (MLH). This will allow to gain insight on the role of atmospheric processing and emission sources on VOC distribution at larger spatial scales. Unfortunately, knowledge of the qualitative and quantitative changes of VOCs and their vertical transport in the atmosphere is limited due to challenging experimental setups.

Currently, there are three methods that have been used to study the vertical atmospheric profile of VOCs. One approach is to sample at the top of tall buildings or towers (Ting et al. 2008; Zhang et al. 2020a) which allows for the use of robust and sensitive equipment, such as online gas chromatographs or PTR-Q-MS for the performance of long-term sampling campaigns at high temporal resolution. However, the infrastructures themselves may be sources of VOCs and the sampling height is limited to the altitude of the tower, which usually is within the MLH. Another method involves the use of aircrafts (Schnitzhofer et al. 2009; Xue et al. 2011; Simpson et al. 2020) which can overcome altitude limitations and collect samples from up to 12 km during continuous measurements. However, this technique is very expensive (Chen et al. 2022), requires more strict permits, and is prone to sample contamination from the aircraft itself. An alternative method is to use tethered balloons (Geng et al. 2020; Greenberg et al. 1999.; Sangiorgi et al. 2011; Spirig et al. 2004; Sun et al. 2018; Wöhrnschimmel et al. 2006; Wu et al. 2020; Zhang et al. 2018). This low-cost approach enables the collection of samples up to 300 m or more with a low risk of sample contamination, although this methodology is limited to the use of lightweight equipment resulting on low-volume sampling. Nevertheless, it also offers flexible sampling location possibilities since it can be easily transported into the field. Therefore, sampling can be performed in urban, industrial, or even in forest environments (Greenberg et al. 1999, 2014; Spirig et al. 2004). However, it may lack time resolution as samples are collected from several minutes to several hours, and only under calm weather conditions. In particular, the vertical distribution of VOCs using this approach has been studied over different urban and industrial sites in China (Ting et al. 2008; Zhang et al. 2018; Geng et al. 2020; Yao et al. 2024), Mexico (Wöhrnschimmel et al. 2006), and in European cities as Milan (Sangiorgi et al. 2011).

The aim of this study is to develop and implement a novel methodology using tethered balloons to characterize the VOC distribution and composition at ground level and above the MLH taking as example an industrial area in Catalonia, which frequently experiences strong temperature inversions. The specific objectives include (i) to investigate the levels and composition of VOCs within the mixing layer, influenced by a complex mixture of sources including traffic emissions, and industrial processes, and (ii) to study changes in VOC concentrations and qualitative composition with altitude above the local inversion layer to evaluate the impact of temperature inversions on VOC levels in the low troposphere. To the best of our knowledge, this will be the first study to assess the vertical composition of VOCs in an industrial site in Spain, providing valuable insights into the frequent poor air quality conditions in these areas. This knowledge can easily be extended to other world regions with similar air quality problems.

## Methodology

### Sampling site

Field sampling was carried out in Catalonia, in the NE of Spain, 18 km from Barcelona (Figure S1). The sampling site (41°32′ 17.1′′ N, 2° 11′ 59.7′′ E, 92 m above sea level) was located in a natural protected area of a municipality that is surrounded by 11 industrial parks and the Mediterranean highway (AP-7), which has an average traffic intensity up to 150.000 vehicles per day. In particular, the sampling site was surrounded by various chemical industrial plants and a train manufacturing industry, among other factories. The intense industrial activity, as well as the traffic associated with the transport of merchandises, may have influence on the air quality of the area. In fact, air quality monitoring stations of the regional authorities identified sites in the areas as top 10 sites in Europe with the highest NO_2_ outdoor concentrations.

### Field campaign

#### Sampling with tethered balloons

**Fig. 1:**
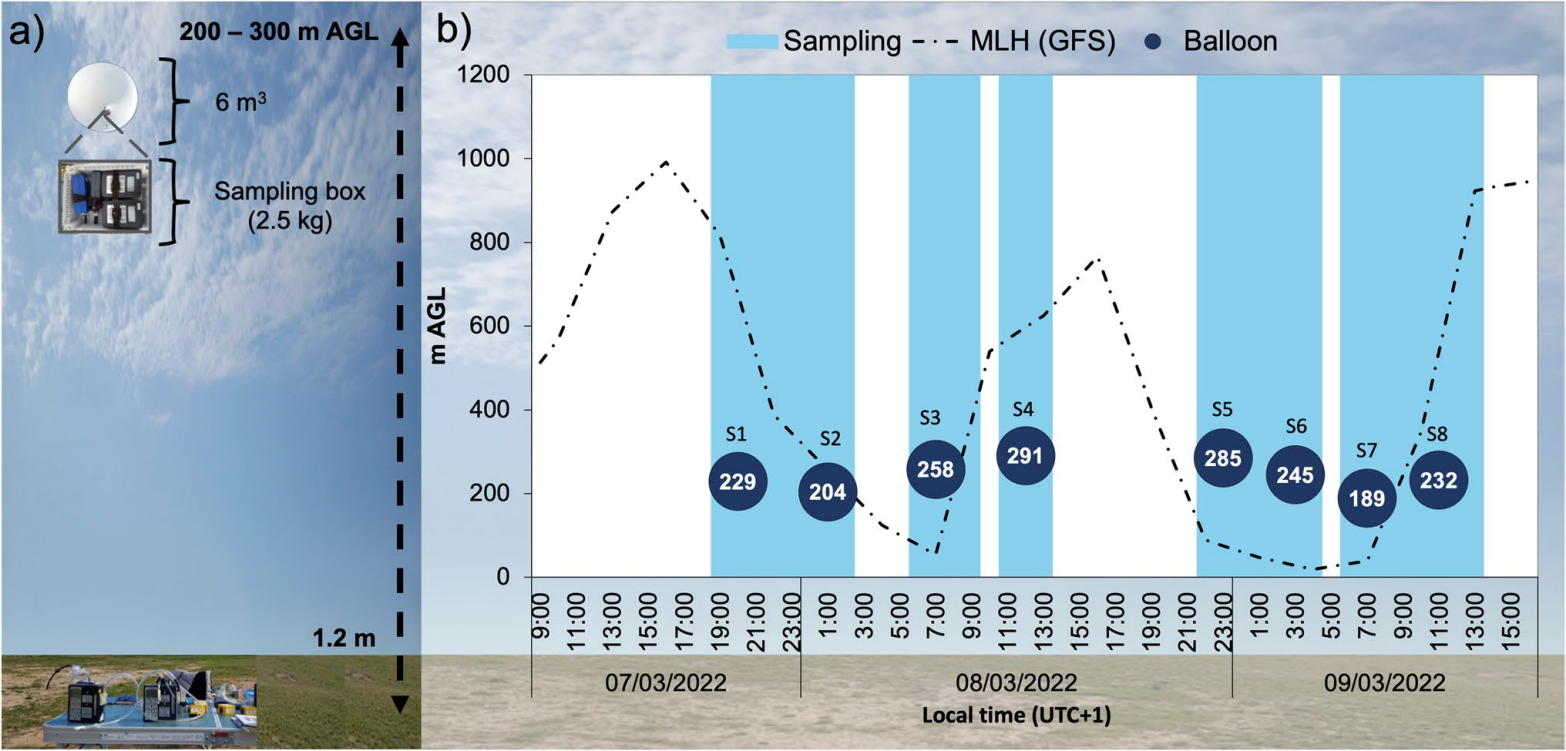
Description of **a** the sampling methodology using tethered balloons and **b** average altitude of the balloon during each sampling period above ground level (AGL) and the mixing layer height (MLH) of the field campaign obtained from the Global Forecast System model (GFS). Date and time are in local time. Light blue background color emphasizes the sampling periods

**Fig. 2:**
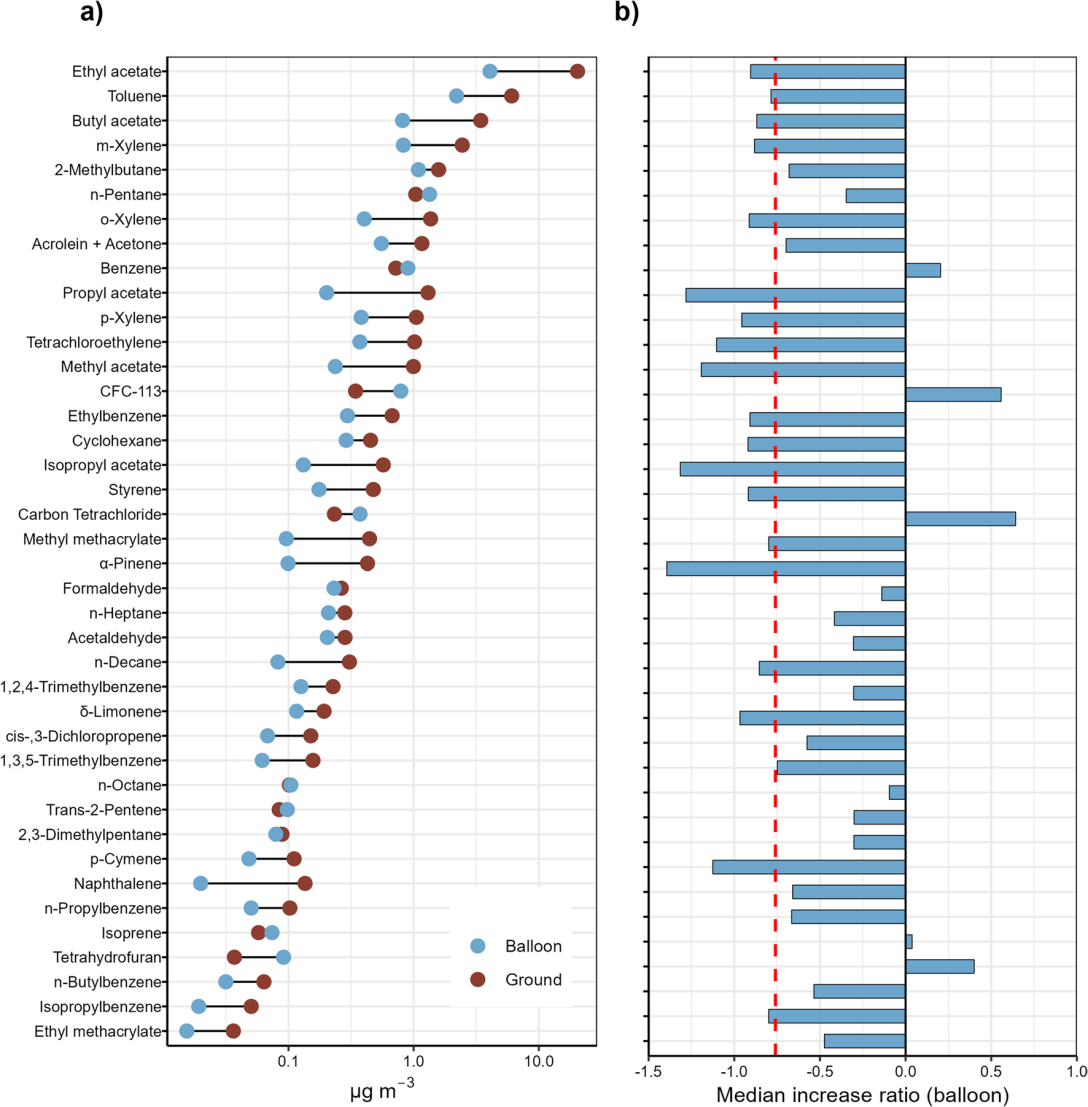
**a** Median concentrations of VOCs at ground and balloon height for the whole field campaign. *X*-axis is presented in log10 scale, **b** median concentration ratio increases at balloon level for each VOC compared to the ground. Dashed red line represent the median TVOCs increase ratio at balloon level

An innovative method was designed to determine the vertical distribution of organic compounds in the low troposphere (Fig. 1a). The method includes the simultaneous air sampling at ground level (1.2 m) and in altitude using tethered balloons. Unlike other studies using meteorological balloons, this method raises sampling devices up to 200–300 m AGL (Fig. 2b) and recover them after each sampling period of 3 h. In addition, it offers the advantage of reducing logistical requirements, as it does not involve transport of heavy equipment and instruments to the field. Due to its lightweight, the tethered balloons require few administrative permits compared to zeppelins or larger balloons, making it a cheaper and a more efficient option for field sampling in altitude.

Balloons were purchased from (The Weather Balloon Mfg. Co., Ltd., Japan) and filled with 3.6 m^3^ of compressed helium (200 bar) in the field up to an inflation diameter of 1.5 m. A box hanging from the balloon housed all the devices needed to sample the gas-phase target compounds. They included (a) a low-flow pump equipped with carbon and Tenax cartridges (Pocket Pump 210-1022, SKC, Eighty-Four, PA, USA), (b) a low-flow pump to work with coated silica cartridges (AirChek XR5000, SKC), (c) a data logger Tinitag Plus (Gemini Data Loggers, Chichester, United Kingdom) for measurement of in situ temperature and relative humidity, and (d) a GPS tracker (SPOT GEN4, Globalstar, Ireland) for accurately measuring the position and altitude of the balloon. All pumps had a quadrupole adjustable low flow tube holder and were compact, portable, and battery-operated. It is important to note that this type of tethered balloon only allows lightweight sampling equipment. Hence, the total weight of the sampling box attached to the balloon never exceeded 2.5 kg. A tether line (Daiwa, J-Braid x 8, France) of 0.142 mm diameter and 500 m length secured the balloon to the ground level platform and a battery-operated winch system (Daiwa Tanacom 1000, Japan) controlled its ascent and descent rates.

To ascertain the composition of VOCs in the lower troposphere of the observation site, a total of eight balloon soundings were conducted from March 7 to March 9, 2022. Each sounding included the collection of two samples representing the VOC composition at ground level and in altitude, respectively. As a result of this, 16 VOC samples were obtained from carbon, TENAX TA, and coated silica cartridges. Details of the physical and meteorological conditions during each vertical profile can be found in Table 1 and Table S1 of the supplementary information (SI).

**Table 1:** Sounding time periods and sampling information: mean balloon sampling height above ground level (AGL) and mixing layer height (MLH), standard deviation (SD), temperature inversion conditions, and total VOCs (TVOCs) concentration at ground and balloon level

Vertical profile code	Starting date	Start-stop time (hh:mm)	Mean balloon height (m AGL)	SD balloon height (m)	Mean MLH (m)	SD MLH (m AGL)	Temperature inversion	TVOCs ground μg/m^3^	TVOCs balloon μg/m^3^
S1	7/3/22	19:15 - 22:20	229	10	602	184	Yes	15.34	10.23
S2	7/3/22	23:10 - 02:15	204	8	278	56	Yes	53.07	29.58
S3	8/3/22	06:00 - 09:08	258	22	181	149	Yes	55.19	15.43
S4	8/3/22	10:08 - 13:00	291	0	583	37	No	83.17	34.82
S5	8/3/22	21:47 - 01:00	285	14	68	19	Yes	27.67	13.74
S6	9/3/22	01:13 - 04:25	245	77	33	12	Yes	43.23	9.56
S7	9/3/22	05:45 - 08:45	189	21	114	100	Yes	59.91	28.60
S8	9/3/22	09:08 - 13:10	232	21	557	276	No	179	43.86

#### VOCs sampling

VOC sampling was achieved using three types of sorbent cartridges, each specific of different groups of compounds. The first was a multi-bed stainless steel tube packed with three graphitised carbon-based sorbents (Sigma-Aldrich, Saint Louis, MO, USA) which was used for the more volatile compounds. These sorbents were arranged from low to high sorption strength and separated by glass wool (150 mg Carbotrap^TM^ C, 150 mg Carbotrap^TM^ and 150 mg Carbotrap^TM^ X). The second was a stainless steel tube filled with 200 mg of TENAX TA (Markes International Ltd, Pontyclun, UK) and was used to sample acetates. Both cartridges were conditioned prior to use with a stream of purified N_2_ at 325°C for 100 min and at 335°C for 15 min in subsequent conditioning cycles using an ATD 400 (Perkin Elmer, Waltham, MA, USA). After conditioning, each cartridge was sealed and stored in a clean container to avoid contamination. The third was a two-bed coated silica cartridge (BPE-DNPH) for the simultaneous measurement of carbonyls and ozone (Uchiyama et al. 2009) in which ozone was trapped by reaction in the first bed containing trans-1,2-bis(pyridyl)ethylene (BPE) to form pyridine-2-aldehyde and the carbonyls were converted to their hydrazone derivatives in the second bed, coated with 2,4-dinitrophenylhydrazine (DNPH).

The carbon and TENAX cartridges were connected in parallel to a low-flow pump using Tygon tubes (Aseptic Group, Limonest, France) and the sampling rate was 55 mL·min^−1^ for 3 hours (9.9 L total air volume). The BPE-DNPH cartridges were wrapped in aluminum foil to be protected from sunlight and were also connected using Tygon tubes to a sample pump operating at a flow rate of 1 L·min^−1^ (180 L total air volume) for 3 h. The flow rates were calibrated in the field for every cartridge at the beginning and the end of each sampling period using calibrated flow meters (TPF Control b.v, The Netherlands; SKC, USA). After sampling, the cartridges were capped and stored separately in refrigerated containers until analysis.

### Laboratory analysis

#### VOC analysis in carbon and TENAX TA cartridges

Collected VOCs were analysed by thermal desorption gas chromatography coupled to mass spectrometry (TD-GC-MS) as described elsewhere (Marco and Grimalt 2015). Briefly, VOCs were thermo-desorbed at 300°C for 5 min with a desorption flow of 40 mL min^−1^ and reconcentrated in a general purpose hydrophobic cold trap at −20°C (U-T2GPH-2S, Markes International). Then, an uncoated and deactivated fused-silica transfer line carried the analytes within a split ratio of 1:5 into a Gas Chromatograph 7890 (GC; Agilent Technologies Inc., Santa Clara, CA, USA) coupled to a mass spectrometer (MS; 5975C Inert XL MSD, Agilent) with an electron impact (EI) ionization source. GC was fitted with a DB-5 MS UI capillary column (60 m × 320 μm × 1 μm; Agilent J&W GC Columns) where compounds were separated at a helium flow rate of 1 mL min^−1^. The oven was programmed to start at 40°C (holding for 10 min), rising to 150°C at 5°C min^−1^, subsequently to 210°C at 15°C min^−1^ and finishing with 10 min of holding time. MS spectra were obtained at 70 eV, being 230ºC and 150°C the temperatures of the ion source and the quadrupole, respectively. EI detector operated in full scan mode and acquired data over a mass range from 30 to 380 a.m.u.

The identification of VOCs was accomplished based on retention times, the ratios of quantifier and qualifier ions and the mass spectra from target compounds in standard solutions. Quantification of the target compounds was carried out by the external standard method. In order to determine linear range and limits of detection, three different calibration solutions with concentrations between 0.25 and 200 μg/mL were prepared in methanol (Merck, Darmstadt, Germany) from the following commercial standards: 8260B MegaMix Calibration Mix (2000 μg mL^−1^ in methanol; Restek, Bellefonte, PA, USA), Cannabis Terpenes Standard #1 (2500 μg mL^−1^ in isopropanol, Restek), 8260B Acetates Mix (2000 μg mL^−1^ in methanol; Restek), FIA Paraffin Standard (Accustandard Inc., New Haven, CT, USA). Each calibration solution was injected (1 μL) into conditioned cartridges through a calibration Solution Ring (CSLRTM, Markes International Ltd, Llantrisant, UK) with N_2_ as carrier gas (flow rate of 50 mL min^−1^) and then was analyzed by TD-GC-MS. The coefficients of determination (*R*^2^) of the calibration curves for target compounds were always >0.99. The instrumental limits of detection (LOD) were determined by multiplying the standard deviation of the response of the calibration curve by 3 and subsequently dividing the result by the slope of the same curve. Method detection limits (MDLs) were obtained by dividing LOD by the sampling volume of each sample. Overall, the MDLs ranged from 0.003 to 0.08 μg m^−3^. To enable comparable and statistically significant analysis, VOC species were chosen if their calculated concentrations in ambient air exceeded the MDLs in more than 75% of the ground samples. Consequently, the final dataset comprised 40 VOCs from various chemical groups. The selected compounds were also found in over 75% of the balloon samples, with the exceptions of ethyl methacrylate and naphthalene, present at 63% of the samples, isopropylbenzene and n-decane at 50%, n-butylbenzene at 38%, and n-octane and isopropyl acetate at only 25%.

#### VOC and O_3_ analysis in BPE-DNPH cartridges

Ozone and carbonyl derivatives trapped in the BPE-DNPH cartridges were eluted with an acetonitrile-water solution (3 mL, 70:30 v/v). Subsequently, these derivatives were analysed on a high-performance liquid chromatograph (Agilent 1200 Series Gradient HPLC, Agilent Technologies) equipped with an analytical column ZORBAX Eclipse Plus C18 Rapid Resolution packed column (150 mm × 4.6 mm, 3.5 μm, Agilent Technologies) at 25°C protected by a guard column ZORBAX Eclipse Plus-C18 (12.5 mm × 4.6 mm, 5 μm, Agilent Technologies) and a diode array detector operating at 360 nm. The analysis was performed at a flow rate of 1 mL min^−1^ lasting 25 min. The mobile phase consisted of A: 5 mmol *L*^−1^ ammonium acetate diluted in water (Merck, Darmstadt, Germany), and B: acetonitrile (Fisher Scientific S.L., Alcobendas, Madrid, Spain) at 55:45, v/v. The injection volume was 10 μL and the sequence of the gradient was: 55% (B) for 4 min, then increased in 12 min to 90% (B) and maintained for 4 min, and finally decreased to 55% (B) to return in 3 min to the initial conditions.

Identification of ozone and carbonyl derivatives was performed by comparing their retention times with standard solutions and quantification by the external standard method. For carbonyls quantification, a calibration solution with concentrations between 0.01 and 3.6 μg mL^−1^ was prepared in acetonitrile (Merck, Darmstadt, Germany) from Aldehydes/Ketones DNPH Standard (15 μg mL^−1^ in acetonitrile; Restek). For pyridine-2-aldehyde-DNPH quantification (ozone derivative), a calibration solution between 0.1 and 300 μg mL^−1^ was prepared in acetonitrile from pyridine-2-aldehyde-DNPH commercial standard (287 ± 9 μg mL^−1^ in acetonitrile, Supelco, USA). Since acrolein and acetone coeluted, they were quantified together as acrolein + acetone. The linearity of the analytical curves (*R*^2^) was always >0.999. MDL detection limits were calculated based on the standard deviation of the response of the low concentration calibration curve and its slope and the sample volume. Overall, MDL ranged from 0.003 to 0.01 μg m^−3^ for carbonyls and from 0.31 to 0.45 μg m^−3^ for ozone.

### Air quality, meteorological, and forecast data

Hourly data of trace gases at ground level during the sampling period were obtained from the nearest automatic monitoring station (AMS) from the Atmospheric Pollution Monitoring and Forecasting Network by the Government of Catalonia (XVPCA: Xarxa de Vigilància i Previsió de la Contaminació Atmosfèrica). Data for NO, NO_x_, NO_2_, SO_2_, and PM_10_ were obtained from the suburban station of Santa Perpetua de Mogoda (41° 31′ 36.2′′ N, 2° 11′ 1.7′′ E) and data for O_3_ from the urban station of Granollers (41° 35′ 55.3′′ N, 2° 17′ 13.6′′ E). Air temperature (°C) and relative humidity (RH %) were recorded by in situ data logger sensors (Tinytag Plus 2) both in the balloon and at ground level. Half-hourly wind speed (m s^−1^), wind direction, and solar radiation (W m^−2^) were obtained at ground level from the Catalan Meteorological Service (SMC) weather station at Parets del Vallès (41° 34′ 2.4′′ N, 2° 13′ 34.3′′ E). Hourly MLH data were obtained from the NCEP Global Forecast System (GFS) with a resolution of 0.25° × 0.25°. In order to assess long-range transport of pollutants, air mass backward trajectories were calculated at 100 m and 500 m AGL by NOAA HYYSPLIT model using data from NCEP GFS system (Stein et al. 2015).

### Statistical analysis

Statistical analysis was performed to evaluate the differences and relationships between VOCs at ground level and at balloon altitude. The Wilcoxon signed-rank test was used to evaluate the differences between paired samples collected in soundings S1 to S8. In addition, Pearson correlation analysis was conducted to evaluate the vertical homogeneity between both levels and the interspecific relationships between different VOCs.

VOCs source apportionment was conducted using Multivariate Curve Resolution–Alternating Least Squares (MCR-ALS) analysis (Tauler 1995) in the MATLAB® environment. This method operates under a non-negativity constraint and, by first requiring a number of components, decomposes the normalized original data matrix into two lower rank matrices: the scores matrix and the loadings matrix. The scores provide information about the contribution of each component to the samples, while the loadings indicate the chemical composition of each component.

## Results and discussion

### Meteorological conditions

The mean air temperature recorded at balloon height was slightly higher than at ground level: 8.8 ± 1.2°C and 7.3 ± 3.5°C, respectively. This difference was consistent with the prevalence of temperature inversion conditions during the observation period. These events occurred in nearly all sampling periods except S4 and S8 (Table 1). In general, the temperature inversion episodes involve accumulation of air pollution near the ground surface by acting as a trap that hinders air convection and subsequent vertical pollutant dispersion (Trinh et al. 2019). It is important to note that the field campaign included rain showers after S4 from 13:00 to 18:30 on 8 March (Figure S2). Unlike temperature, the mean relative humidity (RH %) was homogeneous between the different heights: 81.8 ± 6.8% and 81.5 ± 6.9% at ground and balloon heights, respectively, meaning that sampling conditions during the study were always in high relative humidity. Throughout the field campaign, average daily wind speed ranged from 2.24 m s^−1^ (March 8) to 5.15 m s^−1^ (March 7). Despite the predominance of easterly winds, specific sample periods were affected by winds from the northern east (S2, S5, and S6), east (S3), southeast (S1), west (S7), and southwest (S4 and S8) (Figure S3).

### VOCs concentrations

Forty VOCs from different chemical groups were measured in this study including alkanes, alkenes, aromatics, halogenated and oxygenated hydrocarbons, acetates, and carbonyls. Mean ± standard deviation of these compounds at surface and balloon height, as well as the minimum and maximum concentrations during the studied period are presented in Table 2. Statistical differences between concentrations at ground level and balloon height and the corresponding *p*-values are compiled in Table 2. The total VOCs concentration (TVOCs) was calculated as the sum of the concentrations of the 40 VOCs measured and is shown in Table 1.

**Table 2:** Average concentrations (μg m^−3^), standard deviations (SD) at ground and balloon heights, as well as the minimum and maximum concentrations of 40 VOCs measured throughout the field campaign, and *p*-values from Wilcoxon signed-rank test for assessing the differences in concentrations between ground and balloon heights

Compound	Mean ground	SD ground	Mean balloon	SD balloon	Min	Max	*p-*value
*Alkanes*							
2-Methylbutane	2.74	3.47	2.16	2.96	0.23	11.27	0.250
n-Pentane	4.25	9.40	1.64	1.53	0.04	27.7	0.844
2,3-Dimethylpentane	0.16	0.17	0.08	0.06	< MDL	0.55	0.383
n-Heptane	0.61	0.85	0.27	0.23	< MDL	2.61	0.250
n-Octane	0.18	0.21	0.11	0.08	< MDL	0.68	0.742
n-Decane	0.33	0.29	0.11	0.10	< MDL	0.92	0.039*
Cyclohexane	1.34	2.43	0.39	0.33	0.08	7.32	0.109
*Alkenes*							
trans-2-Pentene	0.16	0.17	0.11	0.08	< MDL	0.56	0.641
Isoprene	0.12	0.17	0.08	0.05	< MDL	0.53	0.742
α-Pinene	0.63	0.66	0.13	0.11	< MDL	1.99	0.016*
δ-Limonene	0.26	0.23	0.11	0.05	< MDL	0.68	0.195
*Aromatics*							
Benzene	0.98	0.84	0.96	0.32	0.19	2.59	1.000
Toluene	6.79	5.38	2.86	2.10	0.69	18.8	0.016*
Ethylbenzene	1.11	1.46	0.31	0.17	0.07	4.60	0.039*
m-Xylene	2.93	2.79	0.90	0.53	0.35	9.47	0.008**
p-Xylene	1.32	1.22	0.41	0.20	0.15	4.19	0.008**
Styrene	0.75	0.66	0.32	0.45	0.06	2.03	0.008**
o-Xylene	2.12	2.98	0.49	0.32	0.18	9.35	0.008**
Isopropylbenzene	0.05	0.04	0.02	0.01	< MDL	0.15	0.008**
n-Propylbenzene	0.12	0.10	0.05	0.02	< MDL	0.34	0.008**
1,3,5-Trimethylbenzene	0.17	0.14	0.06	0.03	< MDL	0.47	0.016*
1,2,4-Trimethylbenzene	0.44	0.64	0.15	0.11	0.03	2.01	0.250
n-Butylbenzene	0.06	0.04	0.03	0.01	< MDL	0.15	0.016*
p-Cymene	0.13	0.07	0.06	0.03	0.04	0.25	0.023*
Naphthalene	0.14	0.08	0.04	0.03	< MDL	0.23	0.016*
*Oxygenated*							
Tetrahydrofuran	0.14	0.26	0.10	0.07	0.01	0.77	0.547
Methyl methacrylate	0.56	0.62	0.11	0.06	0.03	1.85	0.078·
Ethyl methacrylate	0.04	0.03	0.02	0.01	< MDL	0.09	0.078·
*Halogenated*							
Carbon tetrachloride	0.30	0.21	0.42	0.14	0.10	0.60	0.313
CFC-113	0.51	0.31	0.75	0.22	0.25	0.99	0.109
cis-1,3-Dichloropropene	0.17	0.11	0.08	0.04	0.04	0.42	0.016*
Tetrachloroethylene	1.24	0.62	0.35	0.15	0.15	2.25	0.008**
*Acetates*							
Methyl acetate	0.94	0.34	0.29	0.15	0.15	1.31	0.008**
Ethyl acetate	22.5	13.8	6.12	5.17	0.80	40.7	0.016*
Isopropyl acetate	1.11	1.27	0.14	0.15	< MDL	3.41	0.055·
Propyl acetate	1.57	1.31	0.37	0.42	< MDL	3.44	0.008**
Butyl acetate	5.75	6.34	1.60	2.28	0.12	16.4	0.008**
*Carbonyls*							
Formaldehyde	0.34	0.20	0.25	0.09	0.16	0.67	0.078·
Acetaldehyde	0.32	0.17	0.23	0.10	0.09	0.57	0.039*
Acrolein + Acetone	1.19	0.43	0.56	0.10	0.44	1.71	0.008**

Marginally significant (*p*-value <0.1)

^*^Significant (*p*-value <0.05)

^**^Very significant (*p*-value <0.01)

A good correlation (*r* Pearson = 0.85, *p*-value <0.01) between ground level and balloon TVOCs suggests a good correspondence of the concentrations between the two levels. However, the average concentration of TVOCs at ground level was 64.61 ± 50.70 μg m^−3^, ranging from 15.34 to 179.30 μg m^−3^ (Table 1). This was almost three times higher than the mean TVOCs at balloon height (23.23 ± 12.74 μg m^−3^), which ranged from 9.56 to 43.86 μg m^−3^ (Table 1). It is important to note that meteorological events played a key role in the reduction of air pollution during the field campaign. After S4, a rain shower that lasted all afternoon contributed to the reduction of TVOCs at both levels (Figure S2, Table 1), coinciding with the highest MLH (Table 1, Fig. 1b) and the highest average wind speed of the field campaign (Figure S3).

Overall, the general trend in the vertical distribution of VOCs showed a decrease in concentration at the balloon level compared to the ground (Fig. 2a). The median ratio of concentration increases at balloon height to ground level was −0.76, but 19 compounds showed more pronounced decreases (Fig. 2b). Of all the VOCs assessed, 22 compounds had significantly higher median concentrations at ground level than in the balloon (Table 2). These compounds were mainly aromatics, with o-, m-, p-xylenes associated with road traffic (Truc and Kim Oanh 2007; Caselli et al. 2010), halogenated compounds such as tetrachloroethylene, commonly used in industrial dry-cleaning processes in the automotive industry (Zhang et al. 2020b), and methyl, ethyl, propyl, and butyl acetates, which are used as solvents or coatings in paints (Zhang et al. 2020b; Gao et al. 2022). α-Pinene, which is mostly emitted by surface vegetation, and acetaldehyde and acrolein + acetone were also among the compounds with higher concentrations at ground level, which suggest a local primary emission source. This general concentration decrease pattern has also been observed in previous studies conducted with tethered balloons in China (Sun et al. 2018; Zhang et al. 2018; Geng et al. 2020; Wu et al. 2020), Mexico (Wöhrnschimmel et al. 2006), and Italy (Sangiorgi et al. 2011). It has also been observed in studies sampling at the top of high towers at 325 m (Ting et al. 2008) and in the 0.5–5.5-km range using aircrafts (Xue et al. 2011). This difference can be attributed to the vertical dispersion and dilution processes of the primary emitted pollutants (Geng et al. 2020) and to the photochemical degradation processes (He et al. 2023) that take place during transport from the emission sources to the upper parts of the low troposphere.

On the contrary, some VOCs showed an inverse relationship with increasing altitude. Carbon tetrachloride, CFC-113, and tetrahydrofuran showed balloon median concentration ratios that were 1.4–1.5 higher than at ground (Fig. 2b). However, these differences between the two sampling heights were not statistically significant.

This lack of statistical differences in concentrations between the two levels was also observed for most of the alkanes, isoprene, benzene, and 1,2,4-trimethylbenzene (Table 2). These compounds are also associated with road traffic (Tsai et al. 2006; Liu et al. 2008b) but unlike xylenes and other alkylbenzenes, they have lower reactivity rates (Venecek et al. 2018), allowing them to accumulate at higher altitudes once they are released. Since a decrease in concentration with altitude was expected, the similar levels at 200 m could also be explained by either (a) the influence of local plumes or (b) long-range transport of pollutants. Zhang et al. (2018) observed peaks in benzene concentrations at 200–400 m and attributed them to local plume transport. On the other hand, Simpson et al. (2020) investigated the source profile of VOCs over Korea and also attributed the benzene concentrations to long-range transport.

### Qualitative composition of VOCs

**Fig. 3:**
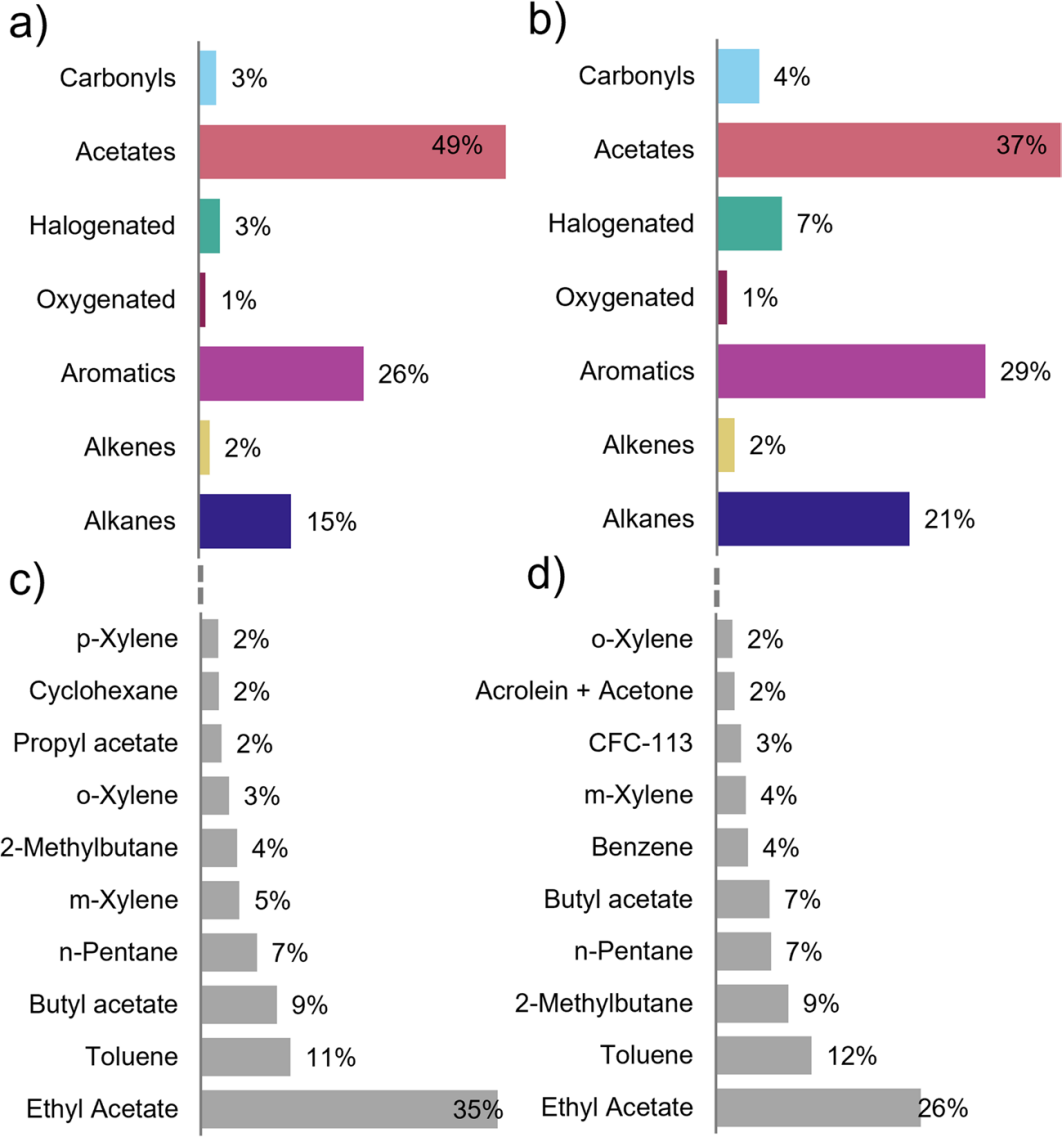
Qualitative composition of VOCs grouped by chemical properties, at **a** ground **b** balloon. Ten most abundant VOCs species at **c** ground level and **d** balloon height

In order to assess the qualitative composition of VOCs at ground level and at balloon height, the contribution of each chemical group was calculated in relation to the mean TVOCs concentration for the entire field campaign at both sampling levels (Fig. 3 a,b).

Despite the different concentration ranges, the VOC composition at both sampling levels was very similar, consistent with the vertical homogeneity observed in the previous section. This result is in line with observations from other studies (Sun et al. 2018; Zhang et al. 2018) and reinforces the hypothesis that local pollution was the main source of the air pollutants up to 300 m AGL at the study site. Acetates and aromatics dominated similarly at both levels, followed by alkanes (Fig. 3a,b). Although not statistically significant, acetates exhibited slightly higher contributions at ground level, while aromatics and alkanes displayed greater dominance at balloon level. Conversely, halogenated and carbonyl VOCs showed significantly higher contributions at balloon height throughout the entire field campaign (*p* <0.05).

During nighttime temperature inversions, a notable increase in the concentrations of halogenated compounds, carbonyls, and alkanes at balloon height compared to ground samples was observed, and the differences were statistically significant (*p* <0.05) for the halogenated compounds and marginally significant (*p* <0.1) for the carbonyls and alkanes (see Figure S4). This difference was also observed by Geng et al. (2020) regarding halogenated compounds and alkanes and was attributed to longer atmospheric lifetimes resulting because of low reactivity (Cox et al. 1976; Venecek et al. 2018). On the other hand, the contribution of acetates at balloon height was significantly lower during these episodes, consistently with the primary origin of these compounds at the study site.

Regarding individual compounds, the 10 VOCs with highest concentrations at both levels were similar. They contributed to 80% of TVOCs at ground level, starting with ethyl acetate and followed by toluene > butyl acetate > n-pentane > m-xylene (Fig. 3c). At balloon height, these ten compounds contributed to 79% of TVOC, with the ranking starting with ethyl acetate, followed by toluene > 2-methylbutane > n-pentane and > butyl acetate (Fig. 3d).

### VOCs and O_3_ within and above the mixing layer

In order to evaluate qualitative differences between samples collected above and below the MLH, the average balloon/ground concentration ratio (B/G) was calculated for each chemical group and sampling period. In this study, VOC samples were obtained over the MLH during four sampling periods: S3, S5, S6, and S7 (Table 1). Besides, the balloon exceeded 50 m the altitude of the MLH during S3 and S7 and 200 m during S5 and S6.

Comparison of B/G ratios show that alkenes, oxygenated compounds, and carbonyls exhibited higher contributions at the balloon when sampling was performed above MLH (Figure S5). However, no statistically significant differences were observed. Within these chemical categories, δ-limonene, α-pinene, tetrahydrofuran, methyl methacrylate, and formaldehyde were identified as the compounds with the highest contribution when balloon was above the MLH. The higher contribution of these VOCs above the MLH could be attributed to the altitude, the time of the day these samples were collected (nighttime) and the specific chemical characteristics of each compound. Photolysis, a key degradation pathway for carbonyls like formaldehyde, is inhibited without sunlight during nighttime sampling. Reactions of δ-limonene and α-pinene with atmospheric oxidants, which usually benefit from sunlight (Atkinson and Arey 2003), are also delayed. Additionally, formaldehyde may have an airborne secondary origin from VOC emissions in other areas and be transported at higher altitudes at the study site through air convection and horizontal circulations. Furthermore, the extended atmospheric lifetime of methyl methacrylate could enable its persistence during the night (Salgado et al. 2011) and its transport from other regions.

**Fig. 4:**
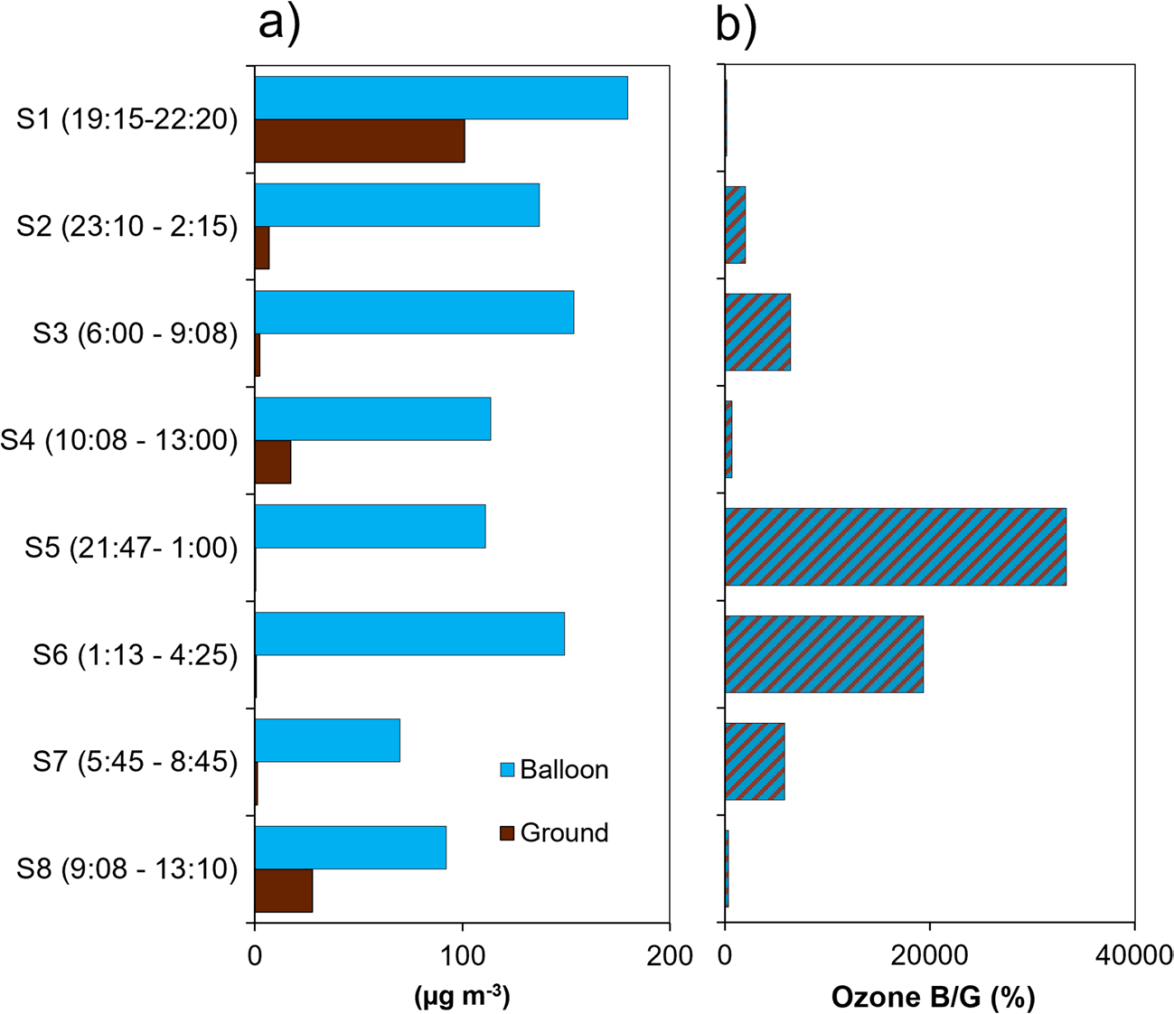
**a** O_3_ concentrations at ground and balloon height; **b** B/G O_3_ concentration ratio (%) for every sampling period of the field campaign

O_3_ concentrations from BPE-DNPH cartridges ranged from 0.77 to 101 μg/m^3^ at ground level and from 69.8 to 180 μg m^−3^ at balloon height. As expected, minimum concentrations at ground level were detected in those nighttime samples when the MLH was the lowest (S3, S5, S6, and S7) and higher concentrations were observed in the afternoon, when the MLH was high and the incidence of solar radiation fostered photochemical reactions between VOCs and atmospheric oxidants (Fig 4a). Particularly, maximum concentrations at both levels coincided during S1 which is an afternoon sample where the MLH was the highest of the whole period. It is important to note that although the balloon was sampling within the MLH in this period, O_3_ concentrations were notably higher at balloon level compared to the ground.

Consistently, ozone concentrations were significantly higher at balloon (*p*-value <0.05) compared to the ground throughout the field campaign. In addition, the balloon-to-ground O_3_ ratio (B/G) was notably and significantly higher (*p* <0.05) in samples collected above the MLH (Fig. 4b) in the night and increased in those periods when balloon sampled >200 m above the MLH (S5 and S6) when a temperature inversion occurs.

Since most of the samples were collected during nighttime in temperature inversion conditions, the increase in O_3_ concentrations at altitude could be explained by the accumulation in the upper troposphere of photochemically formed ozone which does not mix with the layer closest to the ground because of the temperature inversion. Meanwhile, at ground level, residual O_3_ may react with NOx emitted during the day, reducing ozone levels and increasing the B/G ratio (Fig. 4b). When at dawn the solar radiation starts heating the ground’s surface, the inversion layer breaks down and part of the ozone drops down starting new photochemical reactions. Monks et al. (2015) reviewed that in polluted urban regions, the lifetime of ozone in the free troposphere could be enhanced by the higher concentrations of its precursors in the air column. In addition, it is expected that the regional horizontal transport and the injection of O_3_ from the stratosphere could have had an impact on the increase in concentration of this pollutant upper in the troposphere during the field campaign.

### Atmospheric VOC residence time

The atmospheric lifetime of VOCs can range from a few hours to days or even years depending on the photochemical degradation rate of each individual compound and the oxidation capacity of the atmosphere (Atkinson and Arey 2003). In the case of aromatic VOCs such as benzene, toluene, m-xylene, and p-xylene, their respective reaction rates with OH radicals (K_OH_) at 298 K are 1.21, 6.05, 23.6, and 14.3 × 19^−12^ cm^3^ molecule^−1^·s^−1^ (Atkinson 1994), and their approximate lifetime in the atmosphere assuming an OH concentration of 10^6^ rad cm^−3^ are 9.4 days for benzene, 1.9 days for toluene, and 11.8 and 19.4 h for m- and p-xylene (Monod et al. 2001).

**Fig. 5:**
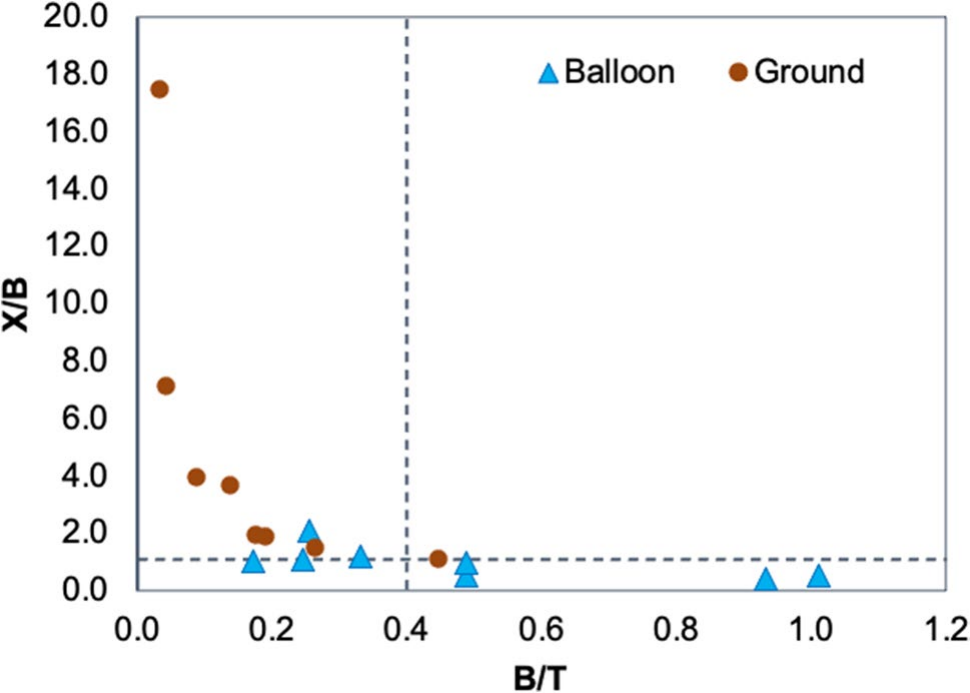
Scatter plots for age evaluation of the air mass at ground and balloon sample heights. Benzene/toluene (B/T) ratios on *x*-axis and m-xylene/benzene (X/B) ratio on *y*-axis

As these compounds typically come from the same sources, but photodegrade differently, they are useful to estimate the residence time of contamination in an air mass. The most commonly used ratios are benzene/toluene (B/T, w/w) and xylene/benzene (X/B, w/w). In particular, an air mass affected by fresh emissions has a B/T less than 0.4 and X/B greater than 1.1, while an aged air mass has B/T higher than 0.4 and X/B less than 1.1 (Liu et al. 2008a; Vo et al. 2018; Geng et al. 2020). In this study, as expected, most of ground samples (Table 3) appeared to be influenced by freshly emitted contaminants (Fig. 5). In particular, samples collected at the morning rush hours (S4 and S8) and in the evening until 2 am (S2 and S5) exhibited X/B higher than 3.5 (Table 3). On the contrary, most of the samples collected at the balloon had values corresponding to aged air parcels, except for two periods, S5 and S8. The former is a sample collected after a rain shower at >300 m above the MLH and under temperature inversion conditions and the latter is a sample collected with deeper MLH which could led to a better diffusion of the pollutants from ground to higher altitudes.

**Table 3:** Intraspecific ratios of m-xylene/benzene (X/B) and benzene/toluene (B/T) for age air mass assessment

Vertical profile code	Starting date	Start-stop time (hh:mm)	Balloon		Ground	
			X/B	B/T	X/B	B/T
S1	7/3/22	19:15 - 22:20	0.37	0.93	1.09	0.45
S2	7/3/22	23:10 - 02:15	0.99	0.17	7.11	0.04
S3	8/3/22	06:00 - 09:08	0.49	0.49	1.94	0.18
S4	8/3/22	10:08 - 13:00	1.04	0.25	17.5	0.03
S5	8/3/22	21:47 - 01:00	1.17	0.33	3.92	0.09
S6	9/3/22	01:13 - 04:25	0.50	1.01	1.86	0.19
S7	9/3/22	05:45 - 08:45	0.93	0.49	1.46	0.26
S8	9/3/22	09:08 - 13:10	2.08	0.26	3.66	0.14

### Identification of the principal VOC sources

To better identify the principal sources affecting the study site at ground and at balloon levels, a source apportionment analysis was performed by applying multivariate analysis (MCR-ALS) techniques to the dataset. This analysis resolved three components that explained 92% of the variance in the samples, and could be interpreted as follows: the first component (C1 *Mix*; explaining 45% of the variance of compounds in the samples) was characterized by overall contributions from most VOCs, with relatively low contributions of acetates and carbonyls. This component represented a mix of different sources, including traffic and industrial emissions. However, the predominance of alkanes and aromatics related to vehicle exhaust, particularly in the diurnal ground sample S8, suggests that traffic could be the dominant contributor for this component. A second component (C2 *Industrial*; explaining 43% of variance) appeared in ground level samples with notable contributions of acetates, tetrachloroethylene, methyl methacrylate, and carbonyl compounds that were related to industrial emissions as mentioned in previous sections. In contrast, this C2 component excluded VOCs related to vehicle exhaust emissions (i.e., n-alkanes) present in the C1 component. A third component (C3 *Aged*; explaining 33% of variance) was more characteristic of the balloon samples and featured stable atmospheric compounds such as CFC-113, carbon tetrachloride, and benzene. Carbonyls, which can have both primary and secondary origins, also showed high contributions in this component. Additionally, the B/T and X/B ratios for the loadings of this third component were 4.4 and 0.06, respectively, while these ratios were 0.8 and 1.5 in the C1 *Mix* and 0.6 and 1.4 in the C2 *Industrial* components. The values in this C3 Aged component corresponded therefore to an aged air mass, while the C1 Mix and C2 Industrial components were more related to source emissions, aligning with the results shown in section 3.5 on B/T and X/B concentration ratios. Previous studies have reported and proved that the B/T concentration ratio is useful for identifying primary sources of VOC emissions (Perry and Gee 1995; Brocco et al. 1997; Barletta et al. 2005, 2008). Benzene is a reliable tracer for biomass and coal combustion and toluene is commonly used in various industrial processes. Barletta et al. (2008) compared these ratios between an urban and an industrial site in the Pearl River Delta region of China and proposed that a B/T below 0.2 can be an indicator of industrial emissions. In the present study, all samples collected at ground level showed concentrations ratios <0.2 except for the samples collected in S1 from 19:15 to 22:20 and S7 from 05:45 to 08:45 am (Table 3). Therefore, the calculated average ( ± SD) of this ratio was 0.17 ± 0.14, suggesting that industrial activities were the prevalent emissions affecting the air quality at ground level. This result is consistent with the composition of *C2 Industrial*, as this component appears in almost all ground samples. On the other hand, it should be noted that traffic emissions also contribute notably to the study area, especially during rush hours, which was reflected in *C1 Mix*, the component appearing most strongly in sample S8.

The mean B/T concentration ratio of the samples collected at balloon height (0.49 ± 0.32) was slightly different from that of the samples collected at ground level. According to Brocco et al. (1997) and Perry and Gee (1995), a B/T ratio around 0.5 indicates influence from traffic emissions, and Barletta et al. (2005) suggested that a B/T ratio greater than 1 can be attributed to other combustion sources, such as coal and biofuel burning. Overall, samples collected inside the MLH (S2, S4, and S8) had a ratio of around 0.2, indicating the influence of nearby industrial activities. On the other hand, samples collected above the MLH (S3, S5, S7) showed an increased influence from vehicular emissions (B/T between 0.33 and 0.49). Additionally, the B/T from S1 and S6 was 0.93 and 1.01, respectively, suggesting a different combustion source from vehicle exhaust. It is important to note that air currents above the MLH can contain a mixture of compounds emitted from various places. As it is mentioned in “Atmospheric VOC residence time,” the balloon samples seem to consist of aged contaminants, those samples with B/T greater than >0.2 can be the result of a mix of compounds emitted by traffic, other combustion sources, or consequence of the oxidation of primary pollutants that were emitted in the previous days, either locally or from other sites.

**Fig. 6:**
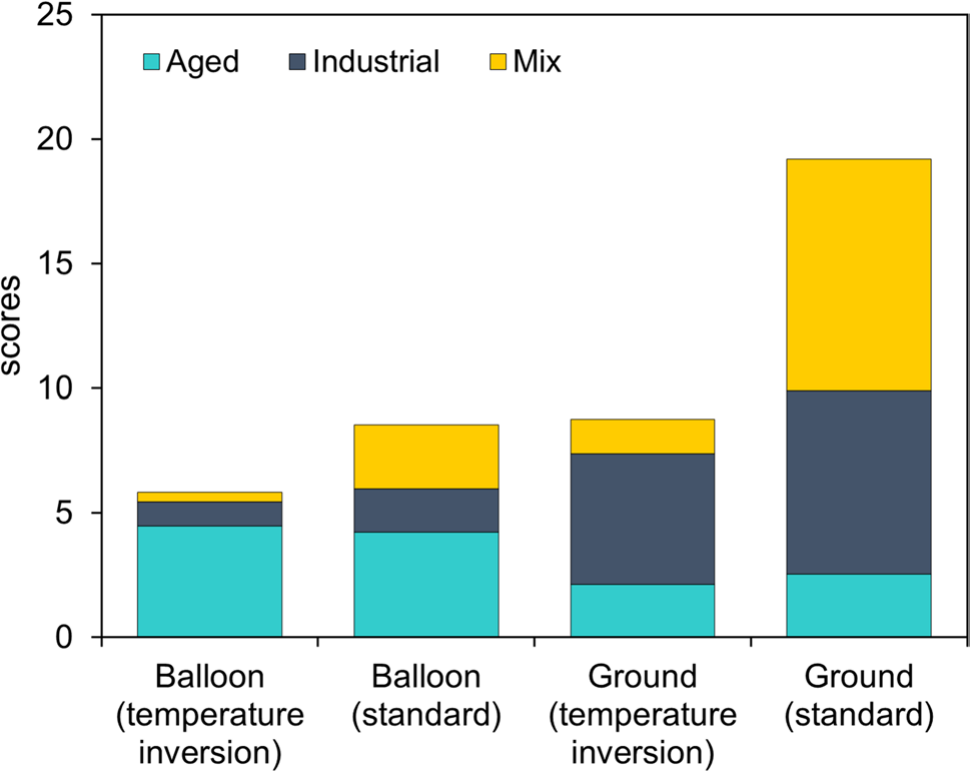
Average scores values for components resolved by the MCR-ALS method applied to ground (G) and balloon (B) samples collected under temperature inversion conditions (inversion) and under standard atmospheric conditions (standard)

The impact of temperature inversions on source apportionment was assessed by comparing the average component scores values in samples collected under inversion and under standard conditions at the different sampling levels. On one hand, Fig. 6 shows that during temperature inversions, balloon samples were predominantly composed of the *C3 Aged* component, while at ground level there was an increase in the influence of the *C2 Industrial* component. On the other hand, under standard conditions, balloon samples encompassed a mixture of the components, indicating vertical mixing processes. Conversely, at ground level, while the values for Aged and Industrial components remained constant, there was a notable increase in the influence of the *C1 Mix* component, which included compounds associated with vehicle exhausts. It is worth noting that the samples collected during temperature inversions were collected at night, while those collected under standard conditions were collected in morning rush hour. The similar scores of the *C2 Industrial* in both situations indicate that the industrial activity did not stop during the night and thus their emissions may accumulate under the temperature inversion layer

Industrial processes release significant quantities of VOCs into the atmosphere influencing local tropospheric chemistry and posing risks to human health (Wang et al. 2023). It is worth noting that 15 out of the 40 measured VOCs in this study are classified as hazardous air pollutants by the US Environmental Protection Agency (EPA) list (www.epa.gov/haps) which includes benzene, toluene, ethylbenzene, styrene, m-xylene, p-xylene, o-xylene, isopropylbenzene, naphthalene, carbon tetrachloride, tetrachloroethylene, cis-1,3-dichloropropene, formaldehyde, acetaldehyde, and acrolein. Most of these hazardous compounds were found at ground level in higher concentrations in the observation site (Table 2) than in other industrial sites across Europe. Specifically, ground-level average concentrations of benzene, toluene, ethylbenzene, m-, p-, and o-xylene (BTEX), as well as naphthalene, were notably higher compared to other industrial sites in Tarragona, Catalonia (Vallecillos et al. 2024), while isopropylbenzene, n-propylbenzene, 1,3,5-trimethylbenzene, and 1,2,4-trimethylbenzene exhibited higher or comparable mean concentrations in Tarragona than in the study site (Vallecillos et al. 2024). Likewise, the concentration of individual BTEX was higher than reported in the Bajio Region in Mexico (Griselda Ceron-Breton et al. 2021) and comparable to levels in different industrial sites in Yokohama, Japan (Tiwari et al. 2010). Furthermore, while benzene and toluene were present in lower concentrations at the observation site, m-, p-, o-xylene, ethylbenzene, and naphthalene were present at higher or comparable levels to those reported in a densely populated industrial zone in Aliaga, Turkey (Dumanoglu et al. 2014) and in an industrial area in Dunkirk, France, during winter (Roukos et al. 2009).

It is important to emphasize that while the reported benzene concentrations are higher than those observed in other industrial sites, they remain below the concentrations documented 18 years ago in March in an urban site located 10 km to the west of the study area (Filella and Peñuelas 2006). In addition, but not less important, current levels are below the annual limit value for the protection of human health (5 μg/m^3^) set by Directive 2000/69/EC.

When comparing to urban ground level concentrations, benzene, toluene, and isoprene concentrations were higher at the observation site than in an urban site in Barcelona (In’t Veld et al. 2024). In contrast, a study conducted in two different urban areas in the Valencian Community found similar levels of xylenes and benzene, but lower levels of toluene than in this study (Galindo et al. 2016). Similarly, a study conducted in Pamplona city reported higher average concentrations of these BTEX compounds (Parra et al. 2009). As expected, our mean BTEX concentrations were consistently higher than the average concentrations recorded at different rural sites in La Ribera of Navarre (Parra et al. 2006) and in Montseny (In’t Veld et al. 2024).

Ethyl acetate emerged as the predominant VOC at both ground and balloon levels, with an average concentration at ground of 22.51 ± 13.77 μg/m^3^ and a peak concentration of 40.73 μg/m^3^ (Table 2) during S4, along with a comparable concentration in S8. This mean ground-level concentration notably exceeds the averages recorded at other industrial sites in Catalonia (Vallecillos et al. 2024) and in a petrochemical industrial area in Yokohama, Japan (Tiwari et al. 2010), surpassing them by nearly 30 and 15 times, respectively. These findings align with prior investigations conducted in China by Wang et al. (2023) and Zheng et al. (2013), which identified ethyl acetate as the most prevalent VOC in industrial processes. Furthermore, it was observed that the tetrachloroethylene concentrations at the study site were similar to those recorded in Japan (Tiwari et al. 2010) but are higher than those found in Turkey (Dumanoglu et al. 2014). Unlike ethyl acetate, the use of this compound is limited to dry cleaning, so its prevalence at industrial sites will depend on the nature of the manufacturing processes carried out.

### Air pollution episodes

Overall, *gaseous* pollutants NO, NO_2_, NO_x_, SO_2_, and PM_10_ measured at the nearest AMS, exhibited similar trends among each other during the field campaign (see Figure S8) and showed an inverse relationship with O_3_. Daily *mean* concentrations for NO ranged from 12.66 to 39.66 μg m^−3^, for NO_2_ ranged from 32.66 to 38.29 μg m^−3^, for NO_x_ from 51.58 to 117 μg m^−3^, for SO_2_ from 1.37 to 1.75 μg m^−3^, for PM_10_ from 19.96 to 24.42 μg m^−3^ and for O_3_ from 18.41 to 54.62 μg m^−3^.

**Fig. 7:**
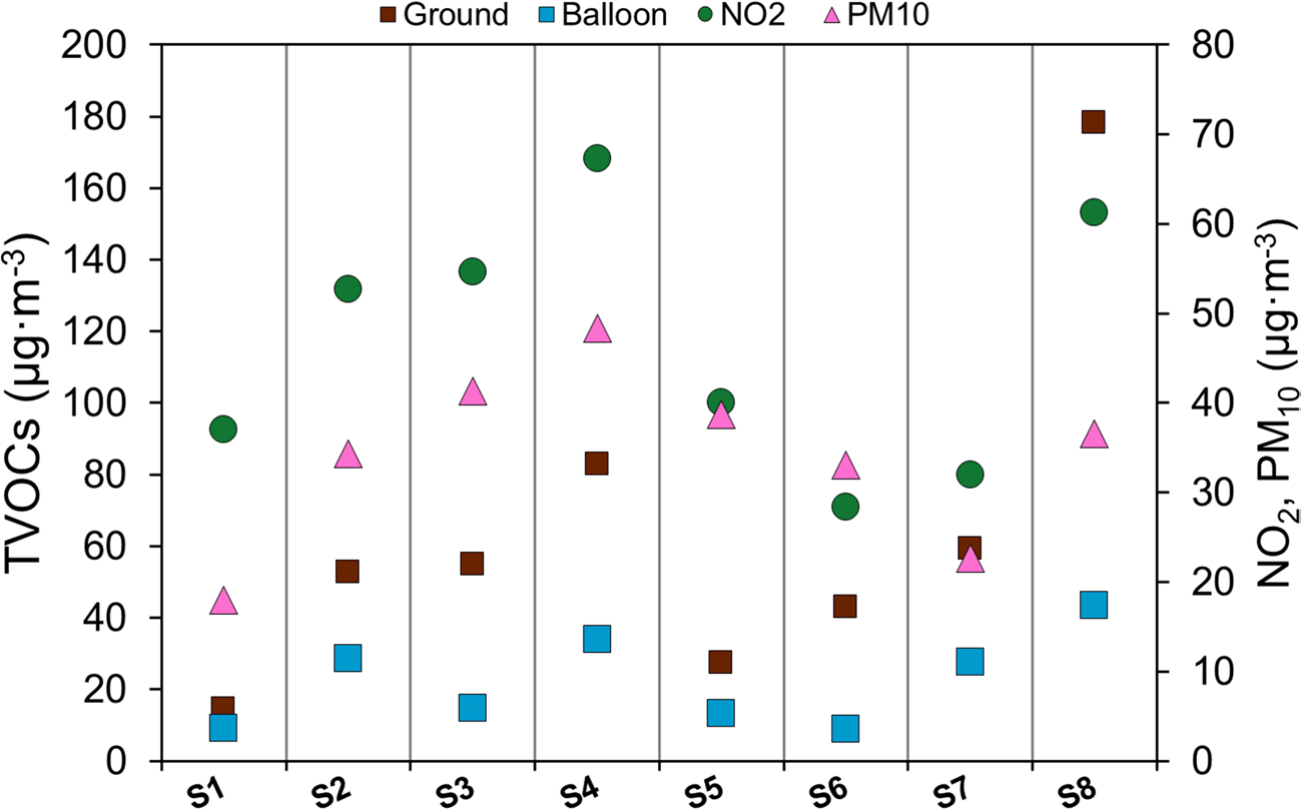
Mean concentrations of TVOCs (ground and balloon), NO_2_ and PM_10_ at ground level calculated for each sampling period during the field campaign. TVOCs concentration is the sum of the 40 VOCs measured

These pollutants (NO, NO_2_, NO_x_, SO_2_, and PM_10_) typically peaked at 8:00 am during morning rush hours, revealing a common source strongly linked to on-road traffic (Fuller and Green 2006; Pérez et al. 2010; McDuffie et al. 2020; Krecl et al. 2021). In these peak periods, high concentrations of NO and NO_2_ resulted in NO_x_ concentrations above 200 μg/m^3^ on March 8 and 9, which exceeded the hourly limit value set by EU Air Quality Directive (2008/EC/50). Furthermore, those peaks coincided with the highest TVOCs concentrations at both sampling heights (Table 1). Specifically, they coincided with the highest concentrations of ethyl acetate and butyl acetate (data not shown) at ground level, among others. Despite most of the VOCs studied herein can be emitted from vehicular traffic (alkanes and aromatics), no association was observed between them and NO, NO_2_, NO_x_, SO_2_, PM_10_ (Table S2). These weak correlations may be explained by the location of the air quality station, which is 1.8 km away from the study site, or by the dominance of industrial activities rather than traffic as main source of VOCs at ground level. However, in Fig. 7, it can be observed that concentrations of TVOCs followed the same pattern as NO_2_ and PM_10_ during the whole field campaign. Furthermore, strong Pearson correlations were observed for oxidized NO_x_ with naphthalene, tetrachloroethylene, and p-cymene at ground level, and secondary pollutants (NO_2_ and PM_10_) with acetates (methyl, butyl, ethyl, acetate) and carbonyls (formaldehyde, acrolein + acetone, and acetaldehyde), indicating a common secondary origin from other primary pollutants (Table S2).

Besides emissions, it should be noted that both peak periods were strongly influenced by southwesterly winds (Figure S3). Backward trajectories for a 24-h period revealed that during peak period S4, air masses at 100 m and 500 m AGL arriving at the study site had passed over Barcelona just a few hours earlier and reached the sampling site through the Besòs river valley (Figure S8); thus, contributions of pollutants transported from the city cannot be ruled out.

In contrast to the other pollutants, O_3_ from AMS peaked after noon, with levels exceeding 75 μg m^−3^ on March 7and 9 after 14:00 when the solar irradiance was as its maximum (Figures S2, S8) and decreased at night. These results agree with measurements in BPE-DNPH cartridges, where higher concentrations of O_3_ at ground level are observed in diurnal samples (Fig. 4a). In addition, this is supported by a strong correlation (*r* Pearson = 0.94, *p*-value <0.001) between both O_3_ levels measured by AMS and in BPE-DNPH cartridges. Atkinson (2000) described that the release of nitrogen oxides, VOCs, and sulfur compounds to the ambient air as the main precursors that initiate chemical chain reactions under sunlight that ultimately lead to the formation of secondary pollutants such as O_3_ in urban areas. At the study site, O_3_ increased when nitrogen oxides (NO_x_ = NO + NO_2_) decreased (Figure S6) and solar irradiance increased (Figure S2). Furthermore, the positive correlation of O_3_ with MLH (*r* Pearson = 0.71, *p*-value >0.05) can be used as an indicator of the diurnal photochemical formation pattern of this pollutant and is consistent with our findings of O_3_ diffusion from higher tropospheric altitudes when temperature inversion breaks down in daylight hours.

## Conclusions

The distribution of VOCs in the low troposphere depends on their emission sources, their photochemical degradation pathways and their atmospheric stability. By employing tethered balloons for sampling, we were able to measure 40 VOCs and O_3_ within and above the MLH. This approach required minimal sampling equipment, avoiding the need for extensive legal permits and logistical arrangements. The study reveals a strong correlation between ground level and balloon TVOCs, suggesting vertical homogeneity in VOC composition up to 300 m. The overall pattern observed in the vertical distribution of VOCs indicated a decline in concentration as the sampling altitude increased by using the tethered balloons. This decrease with altitude denoted the primary origin of VOCs at the study site. On the other hand, compounds with lower reactivity and secondary compounds exhibited similar concentrations at both sampling levels. Qualitative analysis confirmed the similarity in VOC composition at both levels, supporting the hypothesis that local pollution was the primary source of air pollutants up to 300 m AGL.O_3_ concentrations were significantly higher at the balloon’s height, with a greater balloon-to-ground O_3_ ratio in samples taken above the MLH during nighttime temperature inversions. Age air mass assessment, along with source apportionment, indicated that ground samples comprised freshly emitted contaminants from industrial emissions and vehicle exhausts from rush hour traffic. In contrast, balloon samples contained aged contaminants emitted from traffic, industrial activities, and other combustion sources, as well as oxidation of previously emitted pollutants. Ethyl acetate was the predominant VOC at both sampling levels. Despite most VOCs being traffic-related, no significant association with NO, NO_2_, NO_x_, SO_2_, or PM_10_ was observed, likely due to the distance of the air quality station and the dominant influence of industrial activities over traffic emissions at the study site. Further research is needed to overcome the impact of long-range transport of pollutants on local air pollution episodes. In this regard, the use of tethered balloons will help to study the variation in VOC composition at different altitudes, including measurements within and above the MLH.

## Supplementary information

ESM 1 (DOCX 5472 kb)

## Data Availability

Data will be available on reasonable request.
