# Supplementary material for: Measurement of volatile organic compounds using tethered balloons in a polluted industrial site in Catalonia (Spain)
Source: Environ Sci Pollut Res Int. 2024 Jun 26;33(22):11093–109. doi: 10.1007/s11356-024-34020-3 (PMC13415484; doi:10.1007/s11356-024-34020-3)
Supplement: Supplementary file 1 — (DOCX 5472 kb) [file 11356_2024_34020_MOESM1_ESM.docx]

**Supplementary Information (SI) for:**

Measurement of Volatile Organic Compounds using tethered balloons in a polluted industrial site in Catalonia (Spain)

Environmental Sciences and Pollution Research

Isabel Díez-Palet^1,2^, Clara Jaén^1,2^, Esther Marco^1^, Barend L. Van Drooge^1^, Pilar Fernández^1^, and Joan O. Grimalt^1^*

^1^Institute of Environmental Assessment and Water Research (IDAEA-CSIC), Jordi-Girona, 18-26, Barcelona, 08034, Spain

^2^University of Barcelona, Faculty of Chemistry, Martí i Franquès 1-11, 08028-Barcelona, Catalonia, Spain

*Corresponding author:

Joan O. Grimalt

joan.grimalt@idaea.csic.es

Phone: +34934006118

Fax: +34932045904


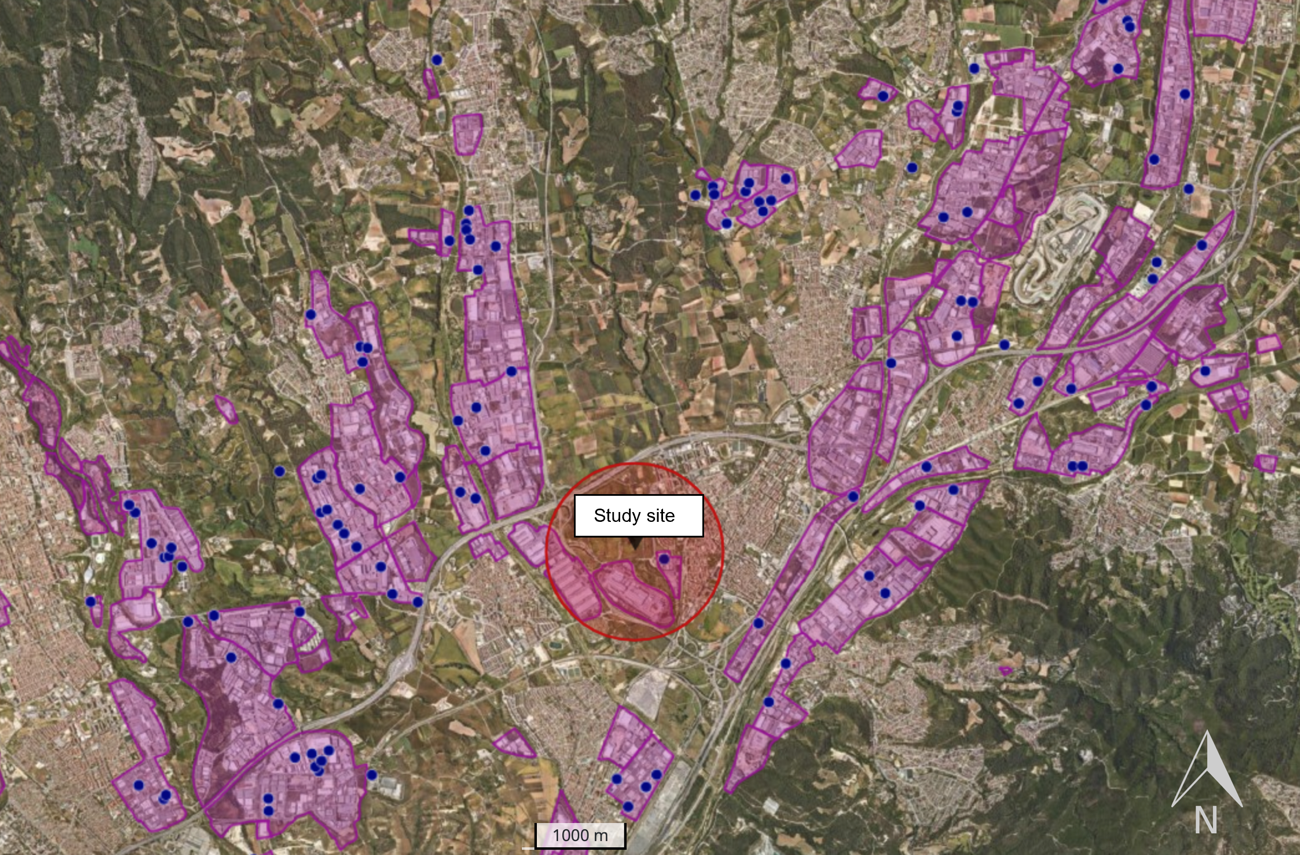


**Fig. S1** Localization of the study site over an industrial area in Catalonia, Spain. A red circumference defines a radius of 1 km from the study site. Industrial polygons and facilities are represented in purple polygons, while facilities authorized to operate for the management of industrial waste are represented in blue points. Sources: Hipermapa of Generalitat de Catalunya (https://sig.gencat.cat/visors/hipermapa.html), Institut Cartogràfic de Catalunya and Agencia de Residus de Catalunya.


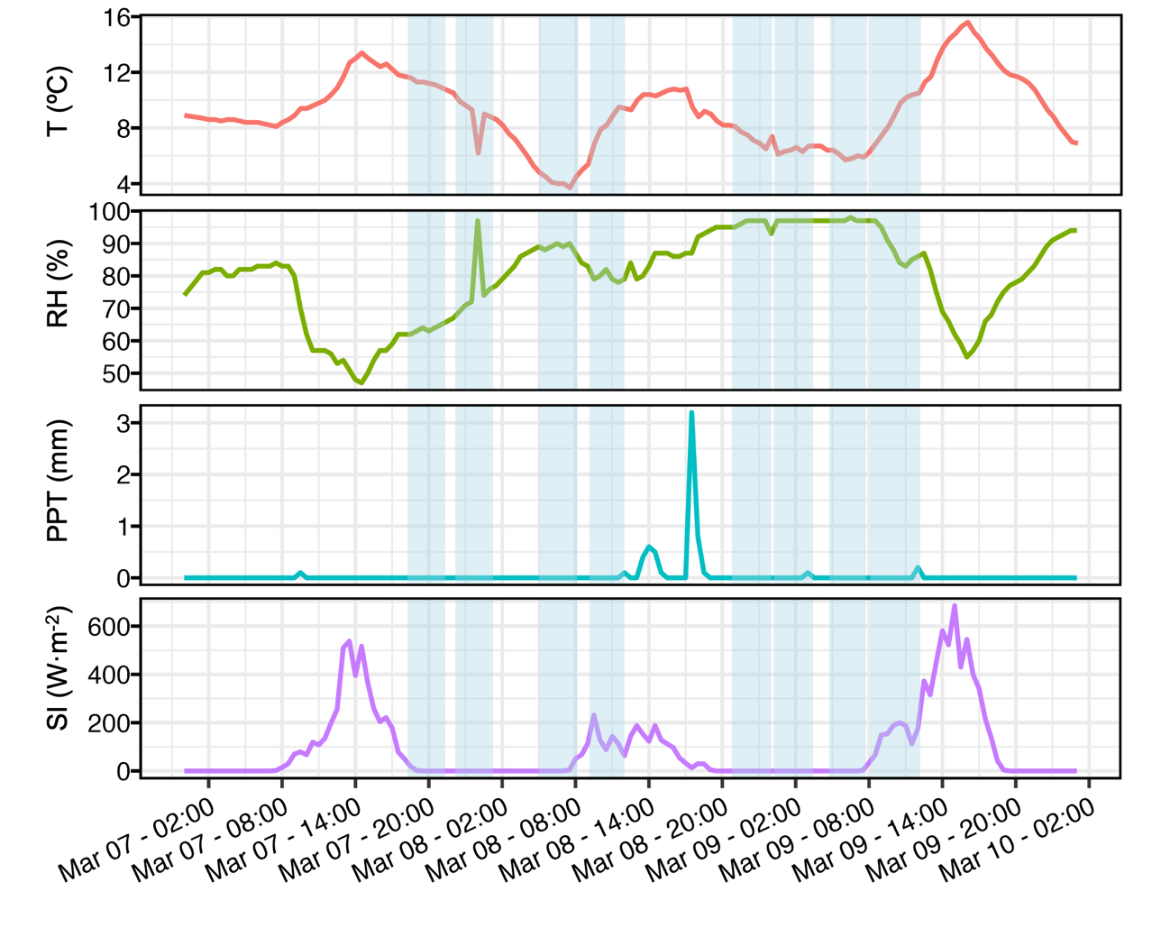


**Fig. S2** Meteorological variables during the sampling campaign in March 2022 in the industrial area. Light blue background color emphasizes the sampling periods.

*
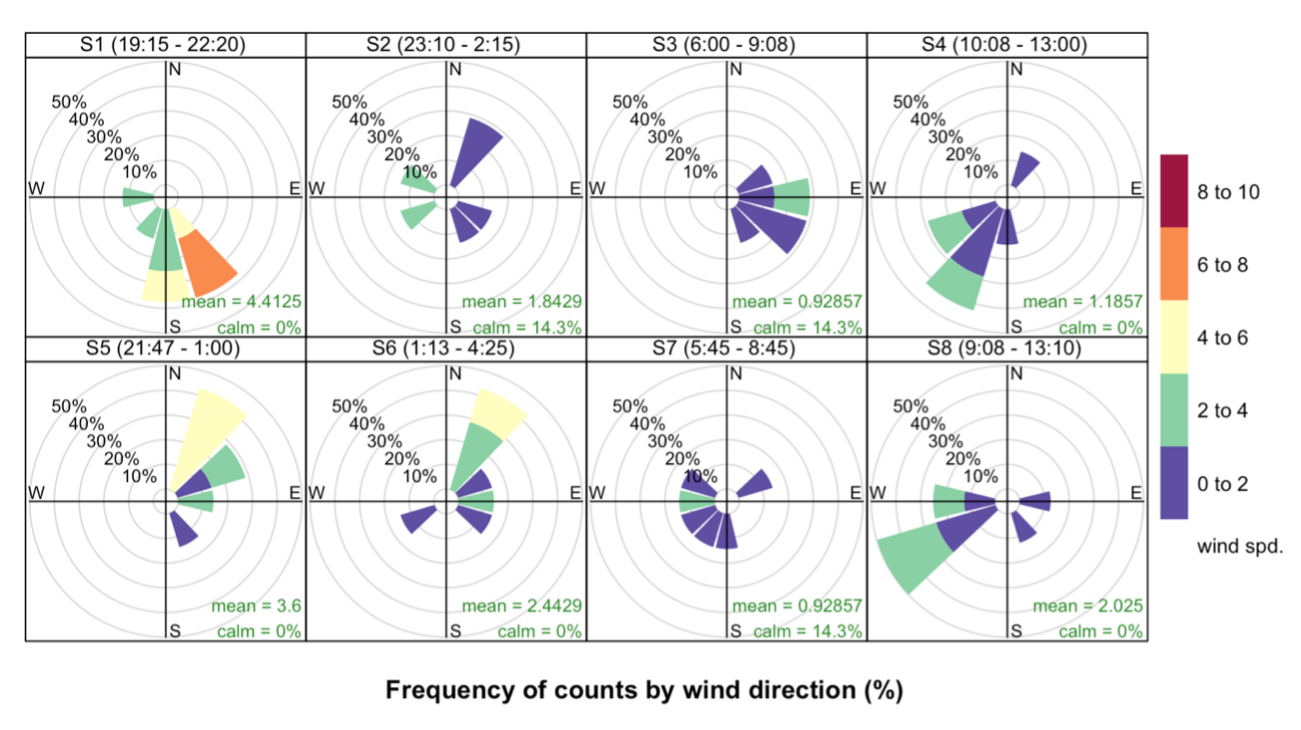
*

**Fig. S3** Average wind speed (m·s^-1^) and wind direction during the sampling periods of the field campaign at the weather station of the Catalonia Meteorological Service (SMC) located near the study site.


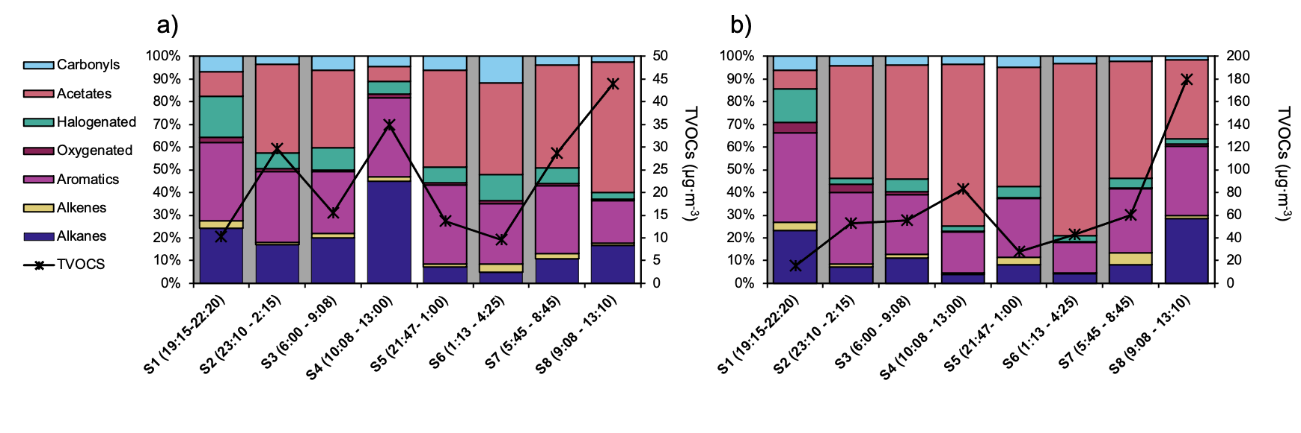
**Fig. S4** Percentage contribution of each category of VOC to Total VOCs concentration (TVOCs) and TVOCs concentration during every sampling period on a) balloon altitude and b) surface. Temperature inversion episodes are represented with grey background color.


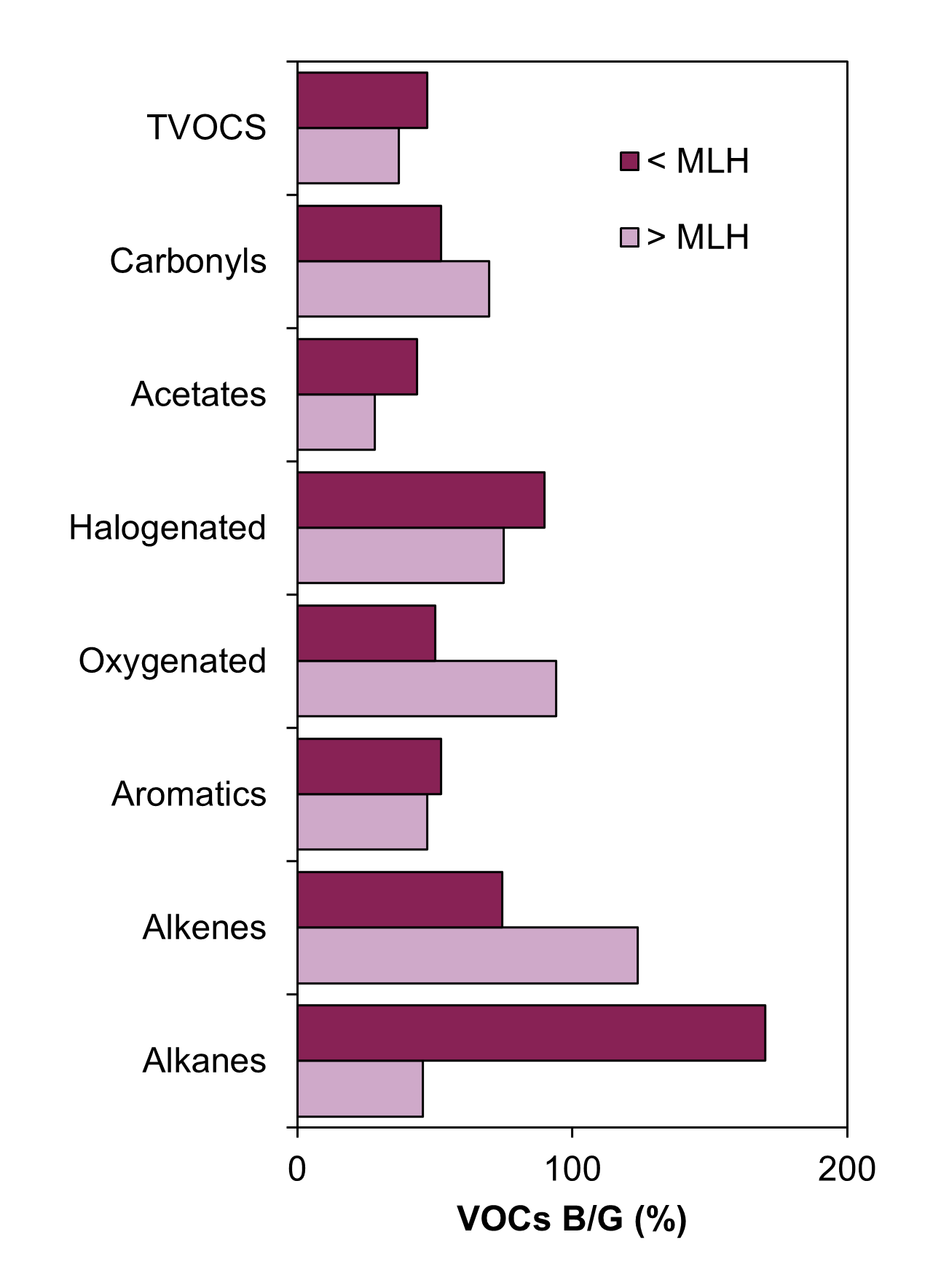


**Fig. S5** Average ground (G) and balloon (B) (B/G) concentration ratio (%) for sampling periods when balloon sampling was performed above the MLH (>MLH) and below the MLH (<MLH).


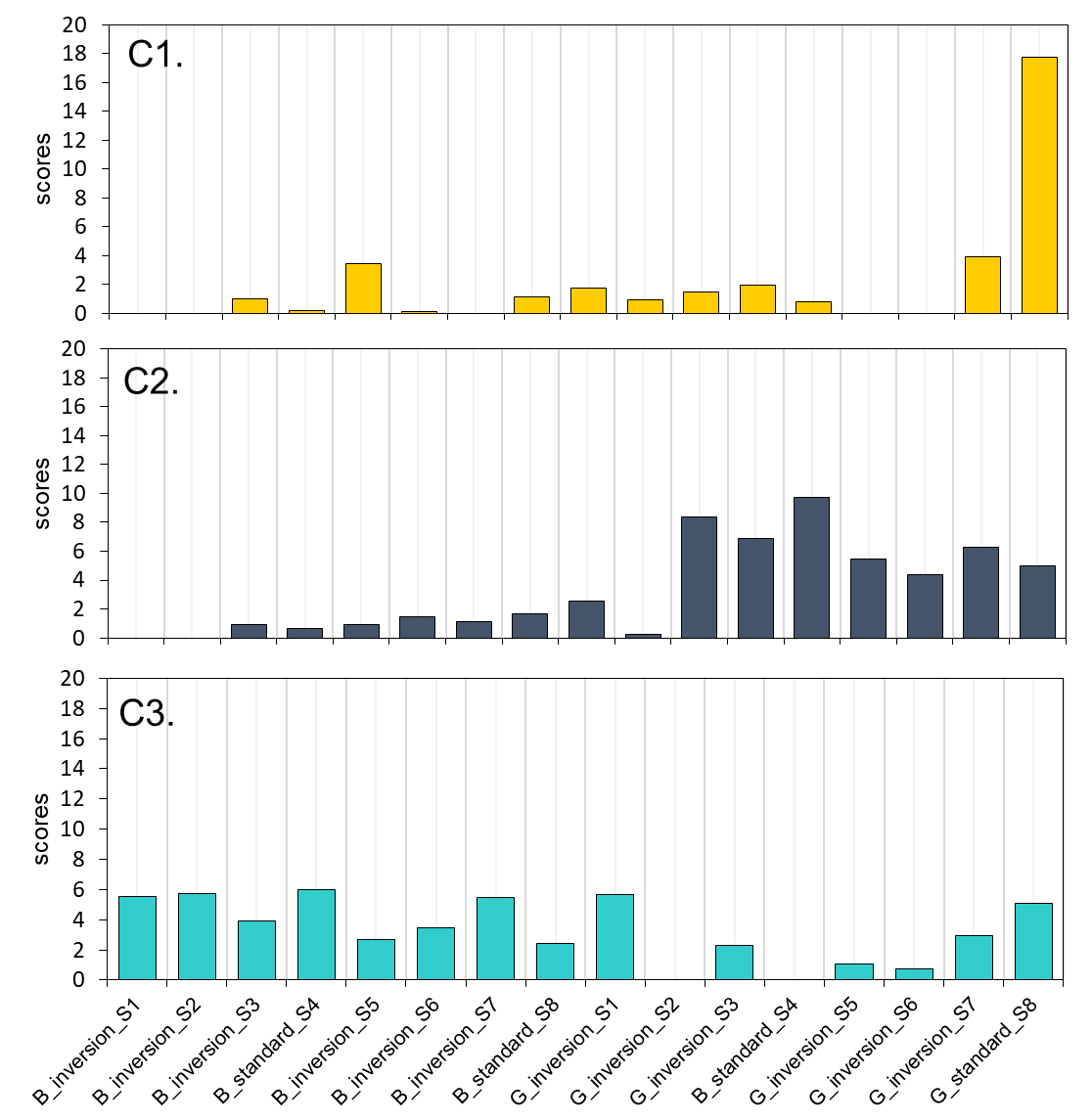


**Fig. S6** Score distribution of the three components resolved from the MCR-ALS analysis in samples collected at the ground (G) and balloon (B) levels from S1 to S8 soundings. Each bar represents the score value of each component in each sample. Samples are labeled as inversion if they were collected under temperature inversion conditions or as standard if they were collected under standard atmospheric conditions.


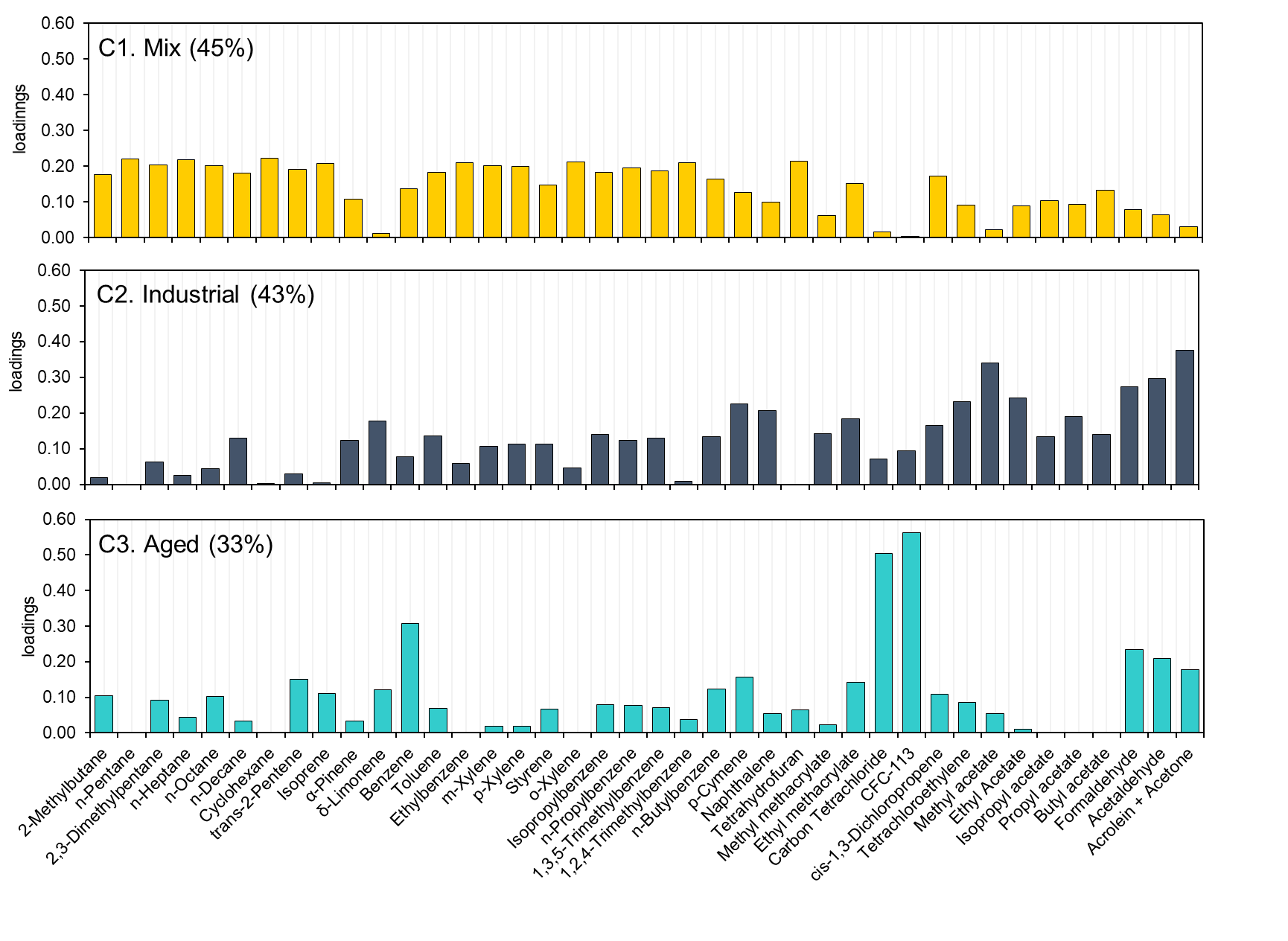


**Fig. S7** Loading distribution of the three components resolved from the MCR-ALS analysis in samples collected at the ground and balloon levels. Each bar represents the loading value of each compound to each component.


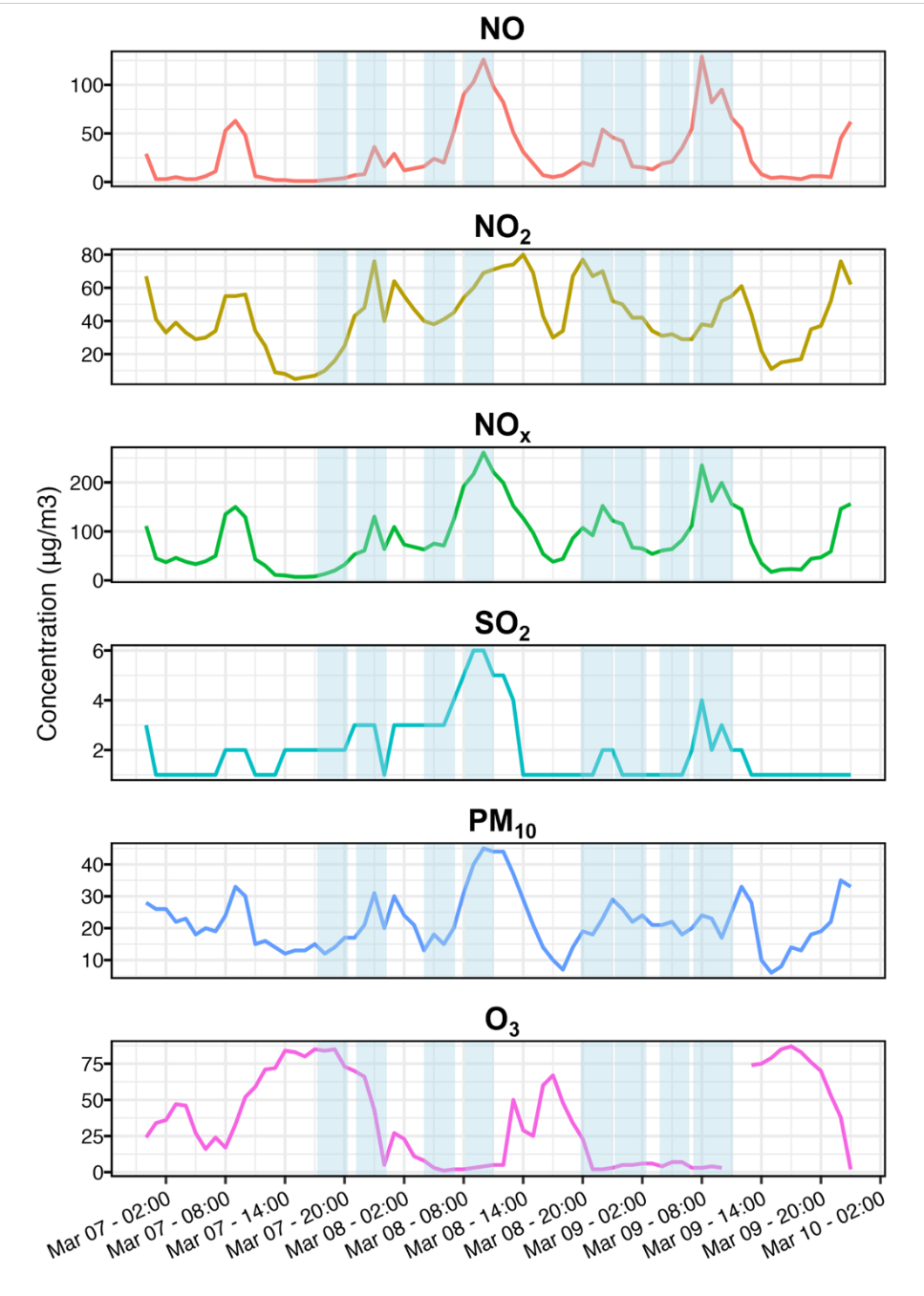


**Fig. S8** Concentration of trace gases during the sampling campaign in March 2022. Light blue background color emphasizes the sampling periods.


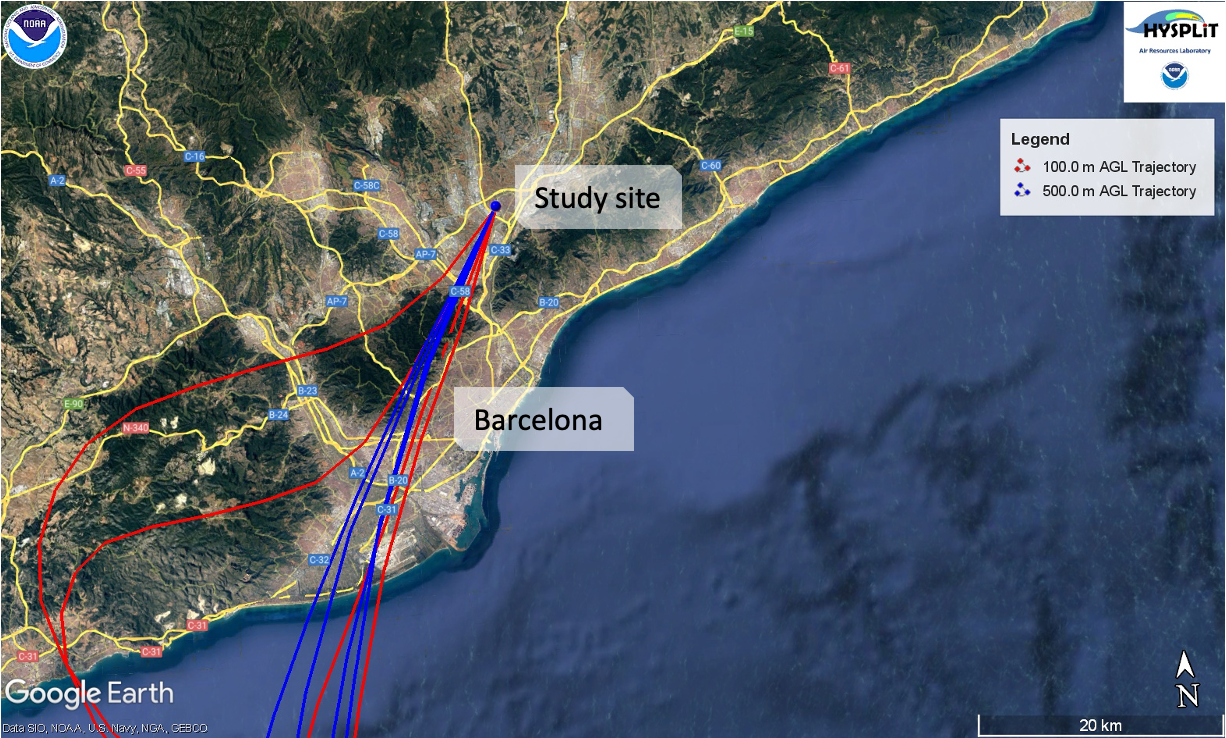


**Fig. S9** Backward trajectories for air masses computed at 100 and 500 m a.g.l by NOAA HYYSPLIT model using data from NCEP GFS system. Data represents the zoomed trajectories of air masses during the sampling period S4 (8 March 10:08 − 13:00 local time) ending at the study site.

| **Table S1.** Average ± standard deviation of temperature (T ºC), relative humidity (RH %) and dew point (DP ºC) of each sample period at ground and balloon level. | | | | | |
| --- | --- | --- | --- | --- | --- |
| Vertical profile code | Starting date | Start - stop time (hh:mm) | T (ºC) | RH (%) | DP (ºC) |
| S1: Balloon | 7/3/22 | 19:15 - 22:20 | 9.11 ± 0.32 | 71.49 ± 1.05 | 4.22 ± 0.25 |
| S2: Balloon | 7/3/22 | 23:10 - 02:15 | 8.60 ± 0.28 | 74.45 ± 1.00 | 4.31 ± 0.13 |
| S3: Balloon | 8/3/22 | 06:00 - 09:08 | 8.15 ± 2.01 | 80.19 ± 9.20 | 4.83 ± 0.39 |
| S4: Balloon | 8/3/22 | 10:08 - 13:00 | 9.64 ± 0.47 | 78.89 ± 3.92 | 6.14 ± 0.65 |
| S5: Balloon | 8/3/22 | 21:47 - 01:00 | 7.88 ± 0.31 | 89.69 ± 3.79 | 6.28 ± 0.38 |
| S6: Balloon | 9/3/22 | 01:13 - 04:25 | 8.05 ± 0.19 | 85.38 ± 2.67 | 5.74 ± 0.32 |
| S7: Balloon | 9/3/22 | 05:45 - 08:45 | 7.43 ± 0.45 | 91.26 ± 2.07 | 6.09 ± 0.18 |
| S8: Balloon | 9/3/22 | 09:08 - 13:10 | 11.24 ± 1.23 | 80.43 ± 3.62 | 7.97 ± 0.69 |
| S1: Ground | 7/3/22 | 19:15 - 22:20 | 8.25 ± 0.21 | 83.92 ± 3.15 | 5.58 ± 0.61 |
| S2: Ground | 7/3/22 | 23:10 - 02:15 | 5.42 ± 0.29 | 96.84 ± 3.28 | 4.95 ± 0.61 |
| S3: Ground | 8/3/22 | 06:00 - 9:08 | 2.51 ± 0.14 | 74.78 ± 49.82 | 2.44 ± 0.21 |
| S4: Ground | 8/3/22 | 10:08 - 13:00 | 10.55 ± 0.20 | 83.54 ± 6.02 | 7.84 ± 0.94 |
| S5: Ground | 8/3/22 | 21:47 - 01:00 | 5.50 ± 0.12 | 76.63 ± 46.75 | 5.52 ± 0.14 |
| S6: Ground | 9/3/22 | 01:13 - 04:25 | 6.43 ± 0.18 | 78.54 ± 42.92 | 6.47 ± 0.21 |
| S7: Ground | 9/3/22 | 05:45 - 08:45 | 5.73 ± 0.11 | 80.15 ± 39.69 | 5.73 ± 0.12 |
| S8: Ground | 9/3/22 | 09:08 - 13:10 | 13.69 ± 0.28 | 79.61 ± 10.36 | 9.90 ± 0.93 |

| **Table S2.** Pearson correlation of VOCs vs pollutants and meteorological variables at ground | **NO** | **NO_2_** | **NO_X_** | **PM_10_** |
| --- | --- | --- | --- | --- |
| **2-Methylbutane** | 0.2 | 0.5 | 0.3 | 0.1 |
| **n-Pentane** | 0.1 | 0.4 | 0.2 | 0.1 |
| **trans-2-Pentene** | 0.2 | 0.3 | 0.2 | -0.2 |
| **Isoprene** | 0.2 | 0.3 | 0.2 | -0.1 |
| **Tetrahydrofuran** | 0.2 | 0.3 | 0.2 | 0.0 |
| **Benzene** | 0.5 | 0.1 | 0.5 | -0.3 |
| **Carbon Tetrachloride** | 0.1 | 0.0 | 0.1 | -0.6 |
| **Cyclohexane** | 0.2 | 0.4 | 0.3 | 0.1 |
| **2.3-Dimethylpentane** | 0.4 | 0.4 | 0.4 | 0.0 |
| **n-Heptane** | 0.3 | 0.4 | 0.4 | 0.0 |
| **Methyl methacrylate** | -0.2 | 0.4 | -0.1 | 0.0 |
| **Toluene** | 0.3 | 0.6 | 0.4 | 0.2 |
| **cis-1-3-Dichloropropene** | 0.3 | 0.6 | 0.4 | 0.2 |
| **Ethyl methacrylate** | 0.6 | 0.6 | 0.7 | 0.4 |
| **n-Octane** | 0.4 | 0.4 | 0.4 | 0.0 |
| **Tetrachloroethylene** | 0.8 | 0.6 | 0.9 | 0.5 |
| **Ethylbenzene** | 0.2 | 0.5 | 0.2 | 0.2 |
| **m-Xylene** | 0.2 | 0.6 | 0.3 | 0.2 |
| **p-Xylene** | 0.2 | 0.6 | 0.3 | 0.2 |
| **Styrene** | 0.4 | 0.2 | 0.4 | 0.0 |
| **o-Xylene** | 0.2 | 0.6 | 0.3 | 0.2 |
| **Isopropylbenzene** | 0.3 | 0.6 | 0.4 | 0.1 |
| **n-Propylbenzene** | 0.3 | 0.5 | 0.4 | 0.1 |
| **1.3.5-Trimethylbenzene** | 0.4 | 0.6 | 0.4 | 0.1 |
| **n-Decane** | 0.5 | 0.5 | 0.5 | 0.2 |
| **1.2.4-Trimethylbenzene** | 0.1 | 0.3 | 0.1 | 0.0 |
| **p-Cymene** | 0.6 | 0.5 | 0.7 | 0.2 |
| **n-Butylbenzene** | 0.3 | 0.6 | 0.3 | 0.0 |
| **Naphthalene** | 0.8 | 0.2 | 0.8 | 0.2 |
| **alpha-Pinene** | 0.5 | -0.1 | 0.5 | -0.3 |
| **d-Limonene** | 0.4 | -0.2 | 0.4 | -0.2 |
| **Methyl acetate** | 0.0 | 0.5 | 0.1 | 0.7 |
| **Ethyl Acetate** | 0.2 | 0.6 | 0.3 | 0.7 |
| **Isopropyl acetate** | 0.2 | 0.1 | 0.2 | -0.2 |
| **Propyl acetate** | -0.1 | 0.2 | 0.0 | 0.0 |
| **Butyl acetate** | 0.3 | 0.8 | 0.4 | 0.5 |
| **Formaldehyde** | 0.1 | 0.9 | 0.3 | 0.7 |
| **Acetaldehyde** | 0.2 | 0.8 | 0.3 | 0.8 |
| **Acrolein + Acetone** | 0.3 | 0.9 | 0.4 | 0.7 |
| **1.1.2-Trichlorotrifluoroethane** | 0.1 | -0.1 | 0.1 | -0.6 |
| **TVOCS** | 0.3 | 0.6 | 0.3 | 0.3 |
| **NO** | 1.0 | 0.2 | 1.0 | 0.2 |
| **NO_2_** | 0.2 | 1.0 | 0.4 | 0.7 |
| **NO_X_** | 1.0 | 0.4 | 1.0 | 0.3 |
| **PM_10_** | 0.2 | 0.7 | 0.3 | 1.0 |
| **T** | -0.3 | 0.4 | -0.2 | 0.1 |
| **RH** | -0.5 | 0.2 | -0.5 | -0.1 |
| **DP** | -0.4 | 0.3 | -0.3 | 0.1 |
